# Downregulation of TLX induces TET3 expression and inhibits glioblastoma stem cell self-renewal and tumorigenesis

**DOI:** 10.1038/ncomms10637

**Published:** 2016-02-03

**Authors:** Qi Cui, Su Yang, Peng Ye, E. Tian, Guoqiang Sun, Jiehua Zhou, Guihua Sun, Xiaoxuan Liu, Chao Chen, Kiyohito Murai, Chunnian Zhao, Krist T. Azizian, Lu Yang, Charles Warden, Xiwei Wu, Massimo D'Apuzzo, Christine Brown, Behnam Badie, Ling Peng, Arthur D. Riggs, John J. Rossi, Yanhong Shi

**Affiliations:** 1 Department of Developmental and Stem Cell Biology, Division of Stem Cell Biology Research, Cancer Center, Beckman Research Institute of City of Hope, 1500 E. Duarte Road, Duarte, California 91010, USA; 2 Irell and Manella Graduate School of Biological Sciences, Beckman Research Institute of City of Hope, 1500 E. Duarte Road, Duarte, California 91010, USA; 3 Department of Molecular and Cellular Biology, Beckman Research Institute of City of Hope, 1500 E. Duarte Road, Duarte, California 91010, USA; 4 Department of Diabetes and Metabolic Diseases Research, Beckman Research Institute of City of Hope, 1500 E. Duarte Road, Duarte, California 91010, USA; 5 Aix-Marseille Université, CNRS, UMR 7325, Centre Interdisciplinaire de Nanoscience de Marseille, 13288 Marseille, France; 6 Integrative Genomics Core, Beckman Research Institute of City of Hope, 1500 E. Duarte Road, Duarte, California 91010, USA; 7 Department of Pathology, Beckman Research Institute of City of Hope, 1500 E. Duarte Road, Duarte, California 91010, USA; 8 Department of Hematology and Hematopoietic Cell Transplantation, Beckman Research Institute of City of Hope, 1500 E. Duarte Road, Duarte, California 91010, USA; 9 Department of Surgery, Beckman Research Institute of City of Hope, 1500 E. Duarte Road, Duarte, California 91010, USA

## Abstract

Glioblastomas have been proposed to be maintained by highly tumorigenic glioblastoma stem cells (GSCs) that are resistant to current therapy. Therefore, targeting GSCs is critical for developing effective therapies for glioblastoma. In this study, we identify the regulatory cascade of the nuclear receptor TLX and the DNA hydroxylase Ten eleven translocation 3 (TET3) as a target for human GSCs. We show that knockdown of TLX expression inhibits human GSC tumorigenicity in mice. Treatment of human GSC-grafted mice with viral vector-delivered TLX shRNA or nanovector-delivered TLX siRNA inhibits tumour development and prolongs survival. Moreover, we identify TET3 as a potent tumour suppressor downstream of TLX to regulate the growth and self-renewal in GSCs. This study identifies the TLX-TET3 axis as a potential therapeutic target for glioblastoma.

## Abstract (web summary)

*TLX* is a nuclear receptor essential for neural stem cell self-renewal and recently involved in glioblastoma development. In this study, the authors show that inhibition of *TLX* expression, achieved using a dendrimer nanovector-delivered siRNAs or viral vector-delivered shRNAs, reduces glioblastoma stem cells self renewal and *in vivo* tumour growth through activation of TET3.

## Graphical abstract

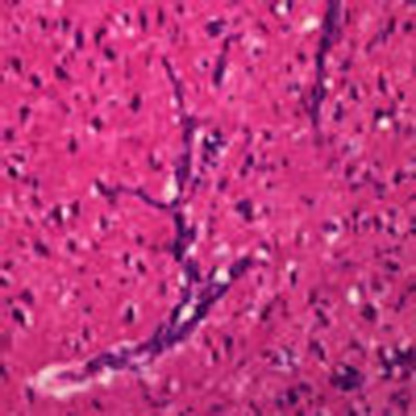

Glioblastoma (GBM) is the most common and aggressive primary brain tumour with median survival time of 14 months after diagnosis^1^. No effective treatment has been developed for GBM patients yet. It has been proposed that GBMs are maintained by a small population of cancer stem cells that retain stem cell properties, are highly tumorigenic and resistant to radiotherapy and chemotherapy^2^,^3^,^4^. The cancer stem cell hypothesis proposes cancer stem cells reside at the top of a cellular hierarchy and have the ability to give rise to the heterogeneous populations of the tumour bulk^5^,^6^,^7^. The presence of cancer stem cells together with the heterogeneity of the tumour mass renders GBM treatment resistant and recurring^8^. Therefore, new therapies are needed to target these cancer stem cells^4^,^9^.

TLX (NR2E1) is a nuclear receptor expressed in vertebrate forebrains^10^ and essential for neural stem cell self-renewal^11^,^12^. Recently, TLX has been shown to be expressed in human GBM tissues and cell lines^13^,^14^, and play a role in GBM development in mouse tumour models^14^. However, the function of TLX in human glioblastoma stem cell (GSC)-initiated tumorigenesis and the effect of modulating TLX expression in human GSCs on the development of GBM remain to be determined.

5-Hydroxymethylcytosine (5hmC) is a form of DNA modification derived from hydroxylation of 5-methylcytosine (5mC). The level of 5hmC is considerably reduced in many types of human cancers^15^,^16^,^17^, including gliomas^18^,^19^,^20^. The level of 5hmC is tightly controlled by the TET family of dioxygenases, which catalyse the conversion of 5mC to 5hmC^21^,^22^. TET proteins have been shown to inhibit haematopoietic transformation^23^,^24^,^25^, breast and prostate cancer invasion and metastasis^26^. However, the role of TET proteins, especially TET3, in GBM tumorigenesis remains largely unknown.

RNA interference holds great promise for tumour therapy. However, efficient delivery of small RNAs *in vivo* represents a major challenge preventing RNA interference from achieving the potency required for successful clinical applications. After ups and downs, RNA interference is now regaining its momentum^27^. Various delivery technologies have been developed for RNA interference. Viral vectors have high delivery efficiency and allow sustained gene silencing with a single injection, offering practical advantage for diseases associated with hard-to-reach organs, such as the brain^27^,^28^. Non-viral vectors, such as cationic lipids and polymers, are developed to increase safety and efficiency of delivery^29^,^30^.

Dendrimers are one of the most promising non-viral vectors for delivering small RNAs by virtue of their well-defined structure and unique multivalent cooperativity alongside the high payload confined within a nanosized volume^31^,^32^,^33^. In particular, ploy(amidoamine) dendrimers bear amine groups at the terminals, which can effectively interact with negatively charged nucleic acids under physiological conditions^34^. They also have tertiary amines in the interior, which can promote the intracellular release of nucleic acids through the ‘proton sponge' effect^35^. However, dendrimer-based delivery of small interfering RNAs (siRNAs) into tumour stem cells is largely unexplored.

In this study, we demonstrate that knockdown of TLX using dendrimer nanovector-delivered synthetic siRNAs or virally expressed short hairpin RNAs (shRNAs) dramatically reduces GSC growth and self-renewal. By transplanting TLX shRNA-transduced GSCs into immunodeficient NOD SCID Gamma (NSG) mice, we show that knockdown of TLX leads to almost complete failure of GSCs to develop tumours in transplanted mouse brains. Furthermore, intratumoral delivery of TLX siRNAs using a dendrimer nanovector or TLX shRNAs using a viral vector inhibits GSC-induced tumorigenesis and prolongs the lifespan of GSC-grafted animals substantially. Moreover, we identify TET3 as a critical TLX downstream target that inhibits GSC self-renewal and tumorigenesis.

## Results

### TLX shRNA reduces GSC self-renewal and tumorigenesis

To determine the role of TLX in human GSCs, we isolated ten primary GSC lines from tumour tissues of newly diagnosed human World Health Organization grade IV GBM patients and cultured them as three-dimensional tumourspheres in a culture condition for GSC enrichment^36^. We classified these GSCs into GBM subtypes using a method previously reported^37^. Among them, PBT003, PBT022, PBT726 and PBT1030 are classical, PBT017, PBT030 and PBT1008 are mesenchymal, whereas PBT024, PBT111 and PBT707 are proneural. These GSCs expressed human neural stem cell markers, Nestin and TLX (Supplementary Fig. 1a). They are also multipotent. When cultured in differentiation condition, they were able to differentiate into βIII tubulin-positive neurons and glial fibrillary acidic protein (GFAP)-positive astrocytes (Supplementary Fig. 1b). After transplantation into NSG mice, these cells could form brain tumours with typical infiltrative features of GBM (Supplementary Fig. 1c).

**Figure 1:**
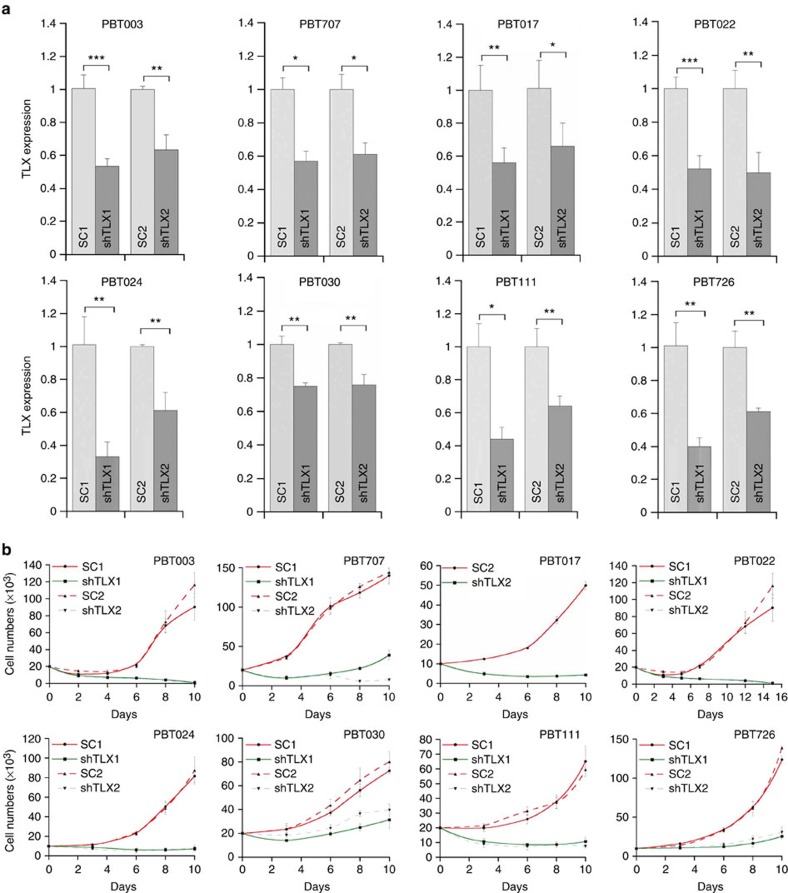
Knocking down TLX expression dramatically reduced the growth of GSCs. (**a**) RT–PCR analysis of TLX expression in GSCs transduced with TLX shRNAs. Scrambled RNAs (SC1 and SC2) were included as negative controls. *N*=3, *P<0.05, **P<0.01, ***P<0.001 by Student's *t*-test. Error bars are s.d. of the mean. (**b**) Growth kinetic analysis of the GSC lines transduced with scrambled control RNAs (SC1, SC2) or TLX shRNAs (shTLX1, shTLX2). *N*=4. Error bars are s.d. of the mean.

To study the function of TLX in GSCs, two shRNAs were designed to knockdown TLX expression in GSCs with two scrambled RNAs as negative controls. After stably transducing TLX shRNA-expressing lentivirus into GSCs, efficient knockdown of TLX was confirmed (Fig. 1a). Knockdown of TLX expression dramatically reduced the growth rate of all GSC lines tested (Fig. 1b), indicating that TLX plays an important role in GSC expansion.

**Figure 2:**
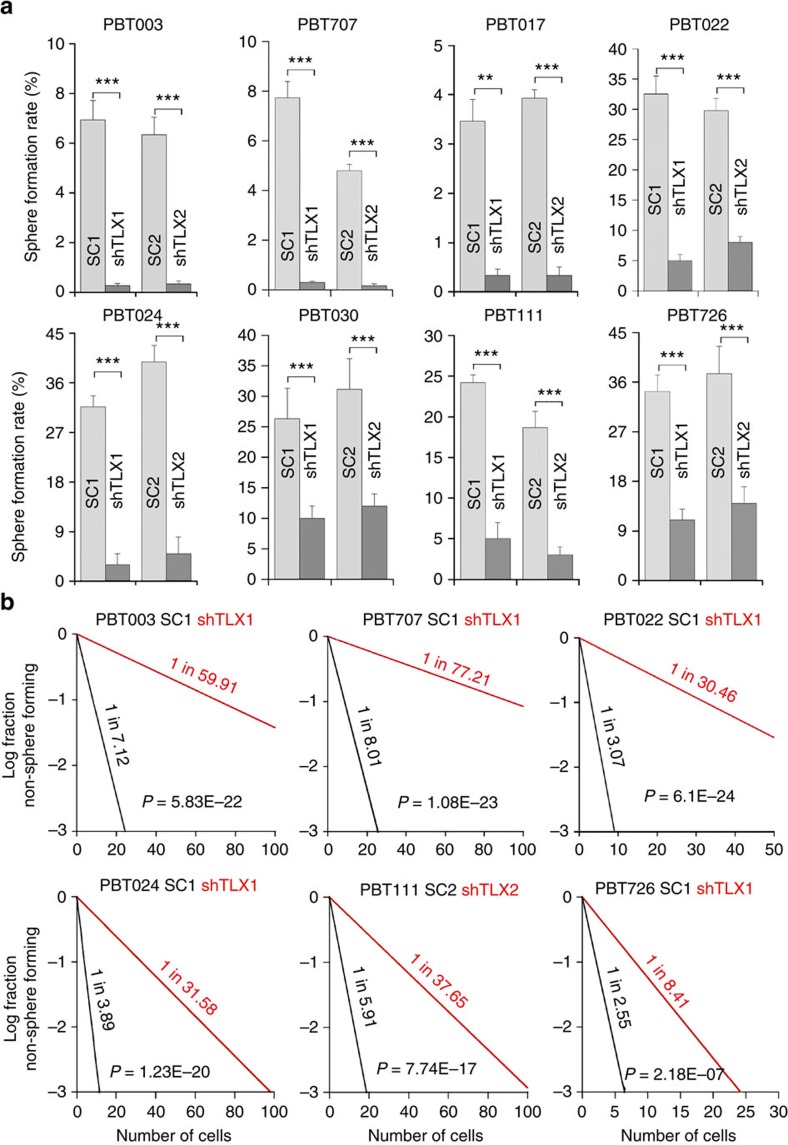
Knocking down TLX expression dramatically reduced the self-renewal of GSCs. (**a**) Quantification of sphere formation rate of GSCs transduced with scrambled control RNAs (SC1, SC2) or TLX shRNAs (shTLX1, shTLX2). *N*=6, **P<0.01, ***P<0.001 by Student's *t*-test. Error bars are s.d. of the mean. (**b**) Limiting dilution assay (LDA) analysis of GSCs transduced with scrambled control RNAs (SC) or TLX shRNAs (shTLX). *N*=20.

We next determined the effect of knocking down TLX on the self-renewal ability of GSCs using clonal analysis and limiting dilution assay. Knockdown of TLX dramatically reduced the self-renewal capacity of GSCs, as revealed by the sharply decreased sphere formation rate and stem cell frequency in TLX shRNA-transduced cells (Fig. 2 and Supplementary Figs 2, 3). Together, these results indicate that TLX is essential for maintaining GSC growth rate and self-renewal ability.

**Figure 3:**
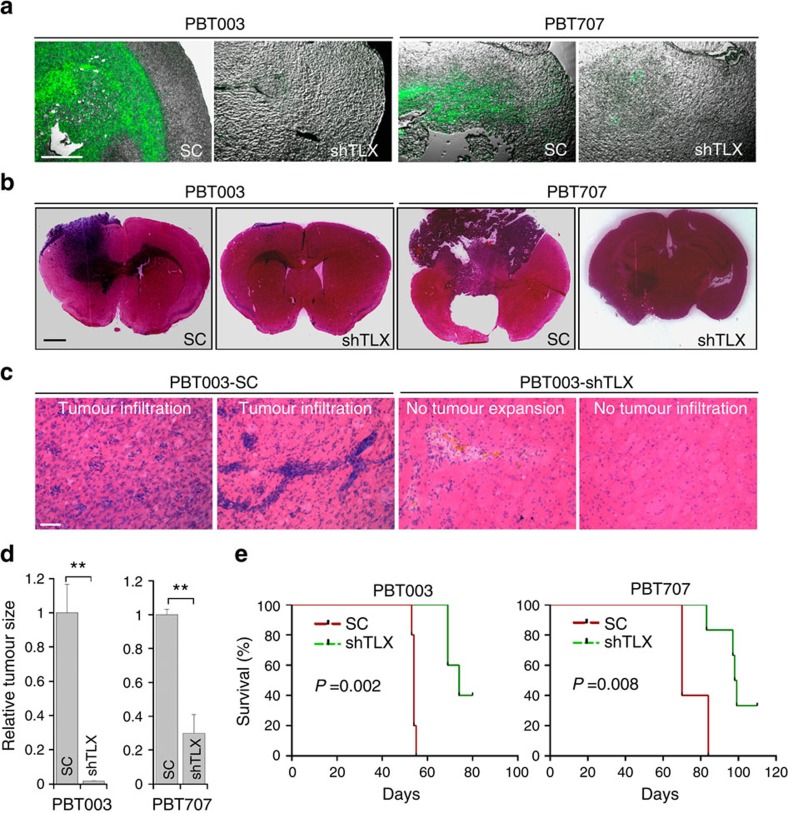
Knocking down TLX reduced the tumour growth and prolonged the survival of tumour-bearing mice. (**a**) GFP fluorescence images of PBT003 cells or PBT707 cells transduced with scrambled control RNAs (SC) or TLX shRNAs (shTLX) and a GFP reporter. Merged images of GFP fluorescence and phase contrast images are shown. (**b**) H&E staining of brain tumour tissues derived from transplanted PBT003 cells and PBT707 cells, transduced with scrambled control RNA (SC) or TLX shRNA (shTLX). (**c**) H&E staining showing typical tumour infiltration characteristics of glioblastoma. (**d**) Relative sizes (volumes) of brain tumours derived from PBT003 or PBT707 cells that were transduced with scrambled control RNA (SC) or TLX shRNA (shTLX). (**e**) Survival curves of NSG mice transplanted with PBT003 or PBT707 cells transduced with scrambled control RNA (SC) or TLX shRNA (shTLX). *X* axis represents days after GSC transplantation. For **a–d**, *N*=4, **P<0.01 by Student's *t*-test. Error bars are s.d. of the mean. For **e**, *N*=5, log-rank test. Scale bar, 200 μm for **a**; 1 mm for **b**; 50 μm for **c**.

The dramatic inhibitory effect of TLX shRNAs on GSC growth and self-renewal *in vitro* prompted us to test whether knockdown of TLX affects the ability of GSCs to form tumours *in vivo*. Two GSC lines, PBT003 and PBT707 cells, were transduced with a lentiviral vector expressing a TLX shRNA and a green fluorescent protein (GFP) reporter. The transduced cells were transplanted into the frontal lobe of NSG mouse brains. Tumour formation and expansion by the TLX shRNA-transduced GSCs were compared with scrambled control RNA-transduced GSCs. Although the same number of GFP-positive cells was injected into each mouse, GFP fluorescence imaging revealed large masses of GFP-positive cells in brains transplanted with control RNA-transduced GSCs, but there was barely any GFP signal in brains transplanted with TLX shRNA-transduced GSCs (Fig. 3a). Haematoxylin and eosin (H&E) staining showed that mice received control GSCs developed large tumour masses with typical infiltrative features of GBM (Fig. 3b,c). Conversely, GSCs treated with TLX shRNAs did not form tumours, or only formed small lesions that were confined to the injection sites (Fig. 3b,c). Stereological measurement of tumour volumes confirmed significantly smaller tumours in brains transplanted with GSCs treated with TLX shRNA, compared with that in brains transplanted with control GSCs (Fig. 3d).

Mice transplanted with TLX shRNA-transduced PBT003 cells had much better survival outcome compared with mice transplanted with scrambled control RNA-transduced cells (Fig. 3e). All mice that were transplanted with TLX shRNA-transduced PBT003 cells survived for more than 70 days post transplant, and 40% of them survived beyond 80 days, whereas all mice that received PBT003 cells transduced with control RNA died before 60 days post transplant. Similarly, all mice that received PBT707 cells transduced with the control RNA died before day 85 post-transplant, whereas most mice that received PBT707 cells transduced with the TLX shRNA survived beyond this point, and 40% survived beyond day 110 (Fig. 3e). Together, these results indicate that knockdown of TLX suppresses tumour growth and increases the lifespan of GSC-grafted mice.

### Knockdown of TLX *in vivo* suppresses tumour progression

**Figure 4:**
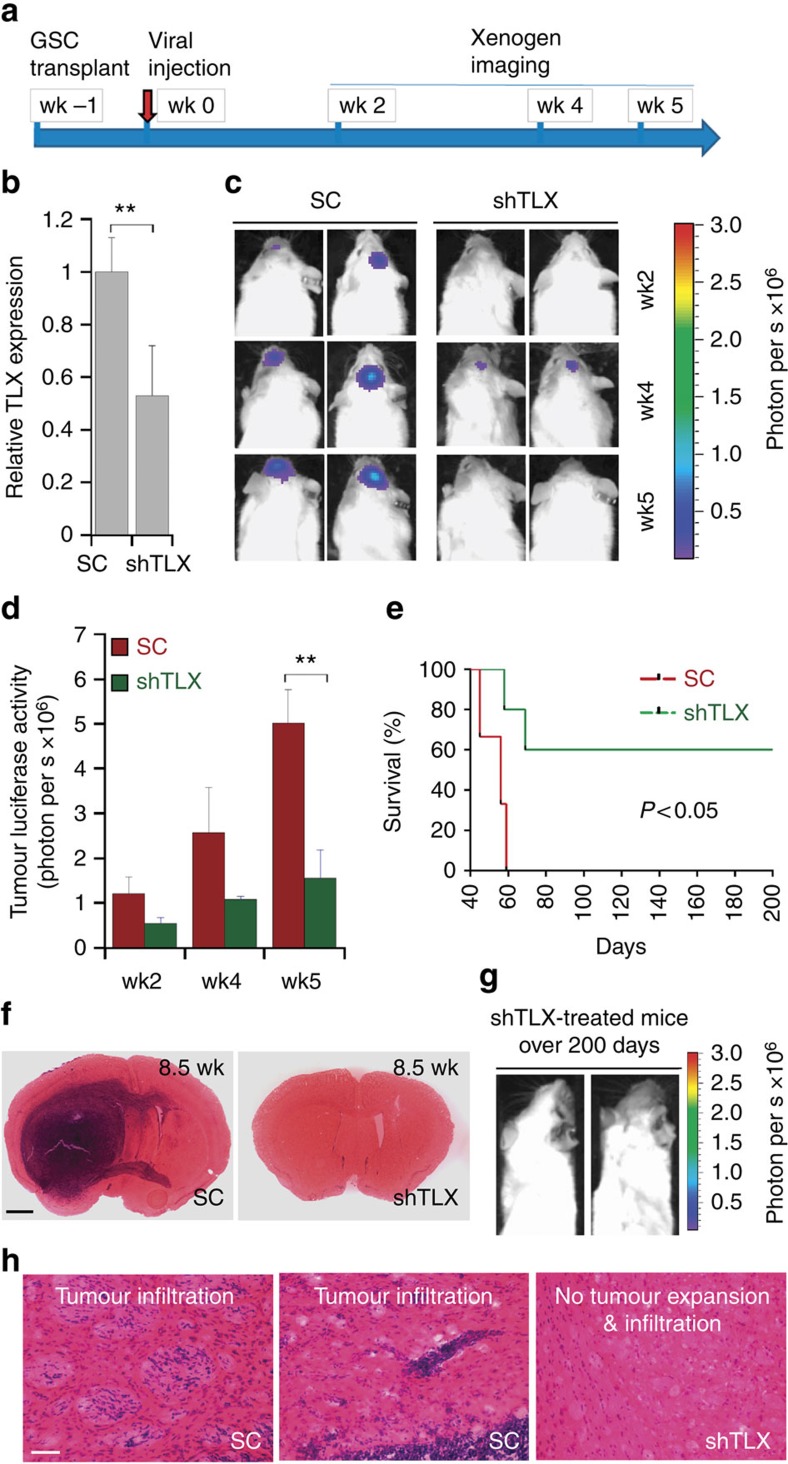
Viral delivery of TLX shRNA inhibits GSC-initiated tumour formation *in vivo* in a xenograft mouse model. (**a**) Schematic of the experimental design, including GSC transplantation, viral treatment and xenogen imaging of xenografted tumours. (**b**) RT–PCR analysis showing TLX knockdown *in vivo*. *N*=3, **P<0.01 by Student's *t*-test. Error bars are s.e. of the mean. (**c**) Xenogen images of brain tumours in NSG mice treated with virus expressing scrambled control (SC) or TLX shRNA (shTLX). The scale for bioluminescence intensity is shown on the right. (**d**). Quantification of the bioluminescence intensity of tumours treated with scrambled control (SC) or TLX shRNA (shTLX) in the brains of engrafted NSG mice. *N*=6, **P<0.01 by Student's *t*-test. Error bars are s.e. of the mean. (**e**) Survival curves of PBT003-engrafted NSG mice treated with virus expressing either scrambled control (SC) or TLX shRNA (shTLX). *X* axis represents days after viral injection. *N*=10 for each treatment group. *P*<0.05 by log-rank test. (**f**) H&E staining of brain tumour tissues derived from transplanted PBT003 cells in NSG mice treated with scrambled control (SC) or TLX shRNA (shTLX). Scale bar, 1 mm. (**g**) Xenogen images of NSG mice survived over 200 days after treatment with virus expressing TLX shRNA (shTLX). (**h**) H&E staining showing typical tumour infiltration characteristics of glioblastoma. Scale bar, 50 μm. wk, week.

Next we tested if knocking down TLX *in vivo* could suppress the progression of human GSC-initiated tumours in a xenograft model. PBT003 cells were transduced with luciferase-expressing lentivirus, which allowed us to monitor tumour growth *in vivo* by bioluminescence imaging. The resultant PBT003 cells were orthotopically transplanted into the frontal lobe of NSG mouse brains to establish tumours. One week after, mice were treated with scrambled control RNA or TLX shRNA-expressing lentivirus by intratumoral injection (Fig. 4a). Knockdown of TLX in PBT003 cells *in vivo* was confirmed by reverse transcription (RT)–PCR using human TLX-specific primers (Fig. 4b). Tumour formation was monitored using bioluminescence xenogen imaging (Fig. 4c). Mice received control RNA-expressing virus developed large tumours, whereas mice treated by TLX shRNA-expressing lentivirus had much smaller tumours (Fig. 4c,d). Bioluminescence measurement showed a significant decrease of tumour signal in mice treated with TLX shRNA-expressing virus at 5 weeks after treatment (Fig. 4d).

Moreover, PBT003-grafted mice treated with TLX shRNA-expressing virus had much better survival outcome compared with mice treated with scrambled control RNA (Fig. 4e). All mice that received control RNA died before day 60 post-treatment and the median survival was 56 days after viral treatment, whereas 60% of mice treated with TLX shRNA survived beyond 200 days post-treatment (Fig. 4e).

When mice in control group died, brain samples were collected for histological analysis. H&E staining revealed the development of big tumour mass and aggressive tumour invasion across the hemisphere in brains of control mice, whereas in brains of TLX shRNA-treated mice collected at the same time, no or much smaller tumour was detected (Fig. 4f–h). The tumours developed in control mice exhibited typical infiltrative features of GBM (Fig. 4h). These results indicate that TLX shRNA-expressing virus suppressed the progression of established tumours and increased the lifespan of treated animals.

To determine the effect of TLX shRNA treatment on tumour progression at a different time point after tumour establishment, we treated mice with control RNA or TLX shRNA-expressing lentivirus 2 weeks after transplantation with luciferase reporter-bearing PBT003 cells (Supplementary Fig. 4a). Bioluminescence imaging showed that TLX shRNA-expressing lentivirus dramatically inhibited tumour growth, compared with control virus (Supplementary Fig. 4b). Bioluminescence measurement showed a significant decrease of tumour size in TLX shRNA-expressing lentivirus-transduced mice, at 3 and 5 weeks after treatment (Supplementary Fig. 4c). These results demonstrate that treatment with TLX shRNA-expressing lentivirus suppressed the progression of established tumours and increased the lifespan of treated mice. The above results together strongly support our hypothesis that TLX could be an effective target to suppress human GSC self-renewal and tumorigenesis.

### The TLX siRNA nanocomplex inhibits GSC tumour progression

In addition to knocking down TLX using a TLX shRNA-expressing viral vector, we explored delivering TLX siRNA oligonucleotides using a non-viral nanovector. We chose the poly(amidoamine) PAMAM dendrimer of generation 5 (referred to as G5 thereafter) to deliver TLX siRNA because it has been shown to deliver siRNAs effectively by forming stable and compact nanoparticles with siRNAs and protect siRNAs from degradation, leading to efficient and long-term gene silencing^34^,^38^. The dendrimer-mediated delivery is also relatively safe without discernible toxicity^39^.

**Figure 5:**
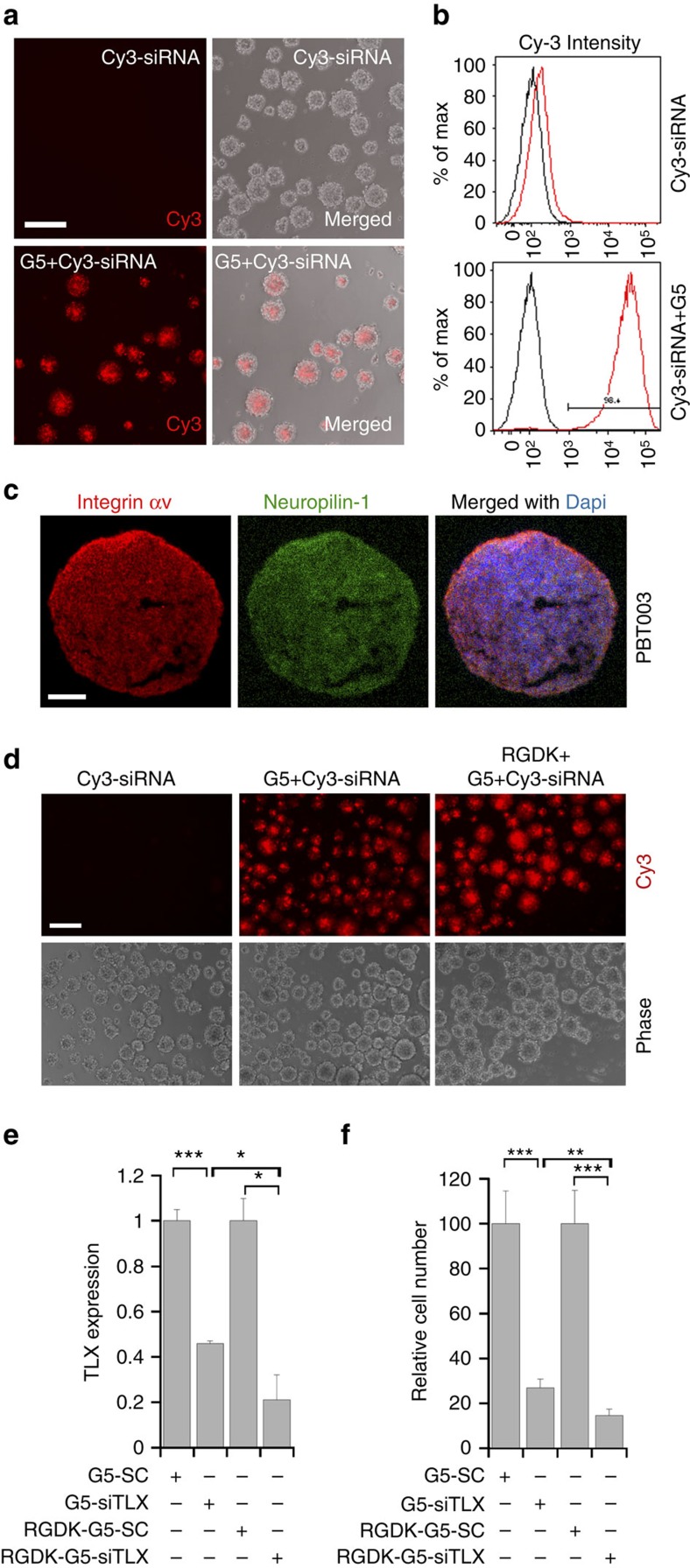
Dendrimer-based nanoparticle delivery of siRNA into GSCs. (**a**) Cellular uptake of Cy3-siRNA delivered by siRNA alone or G5 dendrimers (at N/P ratio of 5) to PBT003 cells. Scale bar, 200 μm. Assays were repeated three times. (**b**) Cellular uptake of Cy3-siRNA analysed by flow cytometry. Uptake of Cy3-siRNA delivered by G5 dendrimer complexes into PBT003 cells was 98.4%. (**c**) Expression of integrin αv and neuropilin-1 on PBT003 GSCs revealed by immunostaining. Scale bar, 100 μm. (**d**) Cellular uptake of Cy3-labelled siRNA delivered by siRNA alone, G5 dendrimers or RGDK-coated G5 dendrimers (at N/P ratio of 5) to PBT003 cells. Scale bar, 200 μm. Assays were repeated three times. (**e**) Knockdown of TLX expression in PBT003 cells by the G5 dendrimer-TLX siRNA complex (G5-siTLX) and RGDK-coated G5-siTLX, analysed by RT–PCR. G5 dendrimer-scrambled control RNA (G5-SC) and RGDK-coated G5-SC nanoparticles were included as controls. (**f**) Growth inhibitory effect of the G5-siTLX and RGDK-G5-siTLX. PBT003 cells treated with G5 dendrimers or RGDK-coated G5 dendrimers complexes with SC or siTLX (at the N/P ratio of 5) were analysed by CellTiter-Glo luminescent assay. *N*=3 for **e**; *N*=6 for **f**. For both **e** and **f**, *P<0.05, **P<0.01, ***P<0.001 by Student's *t*-test. Error bars are s.d. of the mean.

We first tested whether the G5 dendrimer could form stable nanoparticles with siRNAs and deliver siRNAs into GSCs effectively. The G5 dendrimer could form stable complexes with TLX siRNA, as revealed by significant retardation on migration of the G5-siRNA complex at an N/P ratio of 1.0 or above in a gel shift assay (Supplementary Fig. 5a). G5 and TLX siRNA readily formed stable and uniform nanoparticles with an average size slightly smaller than 100 nm in diameter at N/P ratio of 5 (Supplementary Fig. 5b). Consistently, the G5-TLX siRNA complexes were able to protect siRNA from RNase-mediated degradation, whereas naked siRNA was rapidly degraded upon RNase digestion (Supplementary Fig. 5c). When incubated with PBT003 cells, G5 efficiently delivered Cy3-labelled siRNA (Cy3-siRNA) into cells compared with Cy3-siRNA alone control (Fig. 5a). The cellular uptake of G5 delivered Cy3-siRNA was further confirmed by flow cytometry analysis (Fig. 5b).

To achieve tumour cell-targeted delivery, we coated the dendrimer-TLX siRNA nanoparticles with a targeting peptide that contains the dual targeting RGDK motif^40^. The RGD motif directs tumour-specific homing through integrin-dependent binding to tumour cells specifically^41^, whereas the RXXK motif promotes cell and tissue penetration through interaction with neuropilin-1 (refs 41, 42, 43, 44). Both integrin αv and neuropilin-1 were expressed on the surface of PBT003 cells (Fig. 5c and Supplementary Fig. 5d). Decoration of the TLX siRNA-G5 complexes with the RGDK peptide led to the formation of nanoparticles with a size ∼100 nm in diameter (Supplementary Fig. 5e), within a size range required for effective cellular uptake. Coating the G5 dendrimer with the RGDK peptide enhanced the uptake of Cy3-siRNA into GSCs, compared with the uptake of G5 dendrimer-delivered Cy3-siRNA or Cy3-siRNA alone, as revealed by increased intensity of intracellular Cy3 fluorescence (Fig. 5d).

We then treated PBT003 cells with the G5 dendrimer-TLX siRNA nanocomplex with or without RGDK coating. RT–PCR confirmed efficient TLX knockdown by dendrimer-delivered TLX siRNA. Treatment with RGDK-coated dendrimer-TLX siRNA nanocomplex induced even more potent TLX knockdown, presumably due to better cell penetration (Fig. 5e). Compared with control RNA, TLX siRNA delivered by dendrimer dramatically reduced the growth of PBT003 cells, and TLX siRNA delivered by RGDK-coated dendrimer suppressed the growth of PBT003 cells even more (Fig. 5f). Together, these results demonstrated that RGDK-coated dendrimer-TLX siRNA complex efficiently knocked down TLX expression in GSCs and suppressed GSC growth potently.

**Figure 6:**
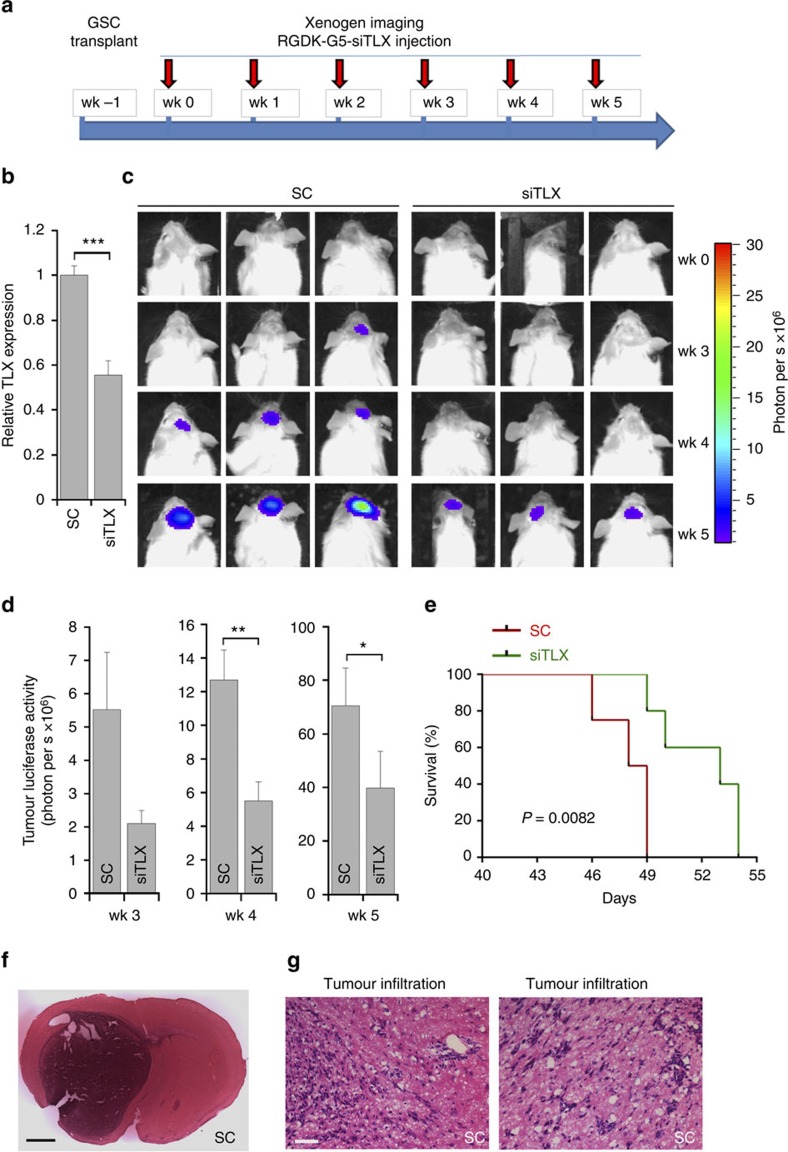
Treatment with the TLX siRNA nanoparticles reduced GSC-derived tumour growth and increased mouse survival. (**a**) Schematic of the experimental design, including GSC transplantation, dendrimer-siTLX complex treatment and xenogen imaging of xenografted tumours. The mice were treated with RGDK-coated G5 dendrimer-siTLX complex or RGDK-coated G5 dendrimer-SC complex. (**b**) *In vivo* knockdown of TLX using RGDK-G5-siTLX nanocomplex. *N*=3, ***P<0.001 by Student's *t*-test. Error bars are s.e. of the mean. (**c**) Xenogen images of brain tumours in NSG mice treated with scrambled control (SC) or TLX siRNA (siTLX). The scale for bioluminescence intensity is shown on the right. (**d**) Quantification of the bioluminescence intensity of tumours treated with scrambled control (SC) or TLX siRNA (siTLX) in the brains of engrafted NSG mice. *N*=7, *P<0.05, **P<0.01 by Student's *t*-test. Error bars are s.e. of the mean. (**e**) Survival curves of PBT003-engrafted NSG mice treated with scrambled control (SC) or TLX siRNA (siTLX). *X* axis represents days after dendrimer-siRNA treatment. *N*=7 for each treatment group. *P*<0.01 by log-rank test. (**f**,**g**) H&E staining showing typical tumour infiltration characteristics of glioblastoma. Scale bar, 1 mm (**f**) and 50 μm (**g**). wk, week.

Next we investigated whether TLX siRNA delivered by RGDK-coated dendrimer could suppress tumour progression in a human GSC-induced xenograft tumour model. PBT003 cells with a luciferase reporter were transplanted into the frontal lobe of NSG mouse brains to establish tumours. One week after transplantation, mice were treated with the RGDK-coated dendrimer-TLX siRNA or dendrimer-control RNA complex by intratumoral injection (Fig. 6a). The *in vivo* TLX knockdown was confirmed by RT–PCR (Fig. 6b). No obvious body weight loss was resulted from surgery or nanoparticle treatment before tumour-induced symptoms developed (Supplementary Fig. 6). Bioluminescence imaging revealed that mice treated with the RGDK-coated dendrimer-TLX siRNA complex had dramatically reduced tumour growth compared with control mice (Fig. 6c). Bioluminescence measurement confirmed that the tumour signals in siRNA complex-treated mice were significantly decreased compared with that in control mice (Fig. 6d). Moreover, treatment with the TLX siRNA complex significantly extended the lifespan of GSC-grafted mice (Fig. 6e). Tumours developed in control mice exhibited typical infiltrative features of GBM (Fig. 6f,g), whereas mice treated with the RGDK-dendrimer-TLX siRNA nanoparticles developed smaller tumour. These results indicate that RGDK-coated dendrimer-delivered TLX siRNA could effectively decrease tumour growth and increase the lifespan of tumour-bearing mice.

### TET3 suppresses GSC self-renewal and tumorigenicity

**Figure 7:**
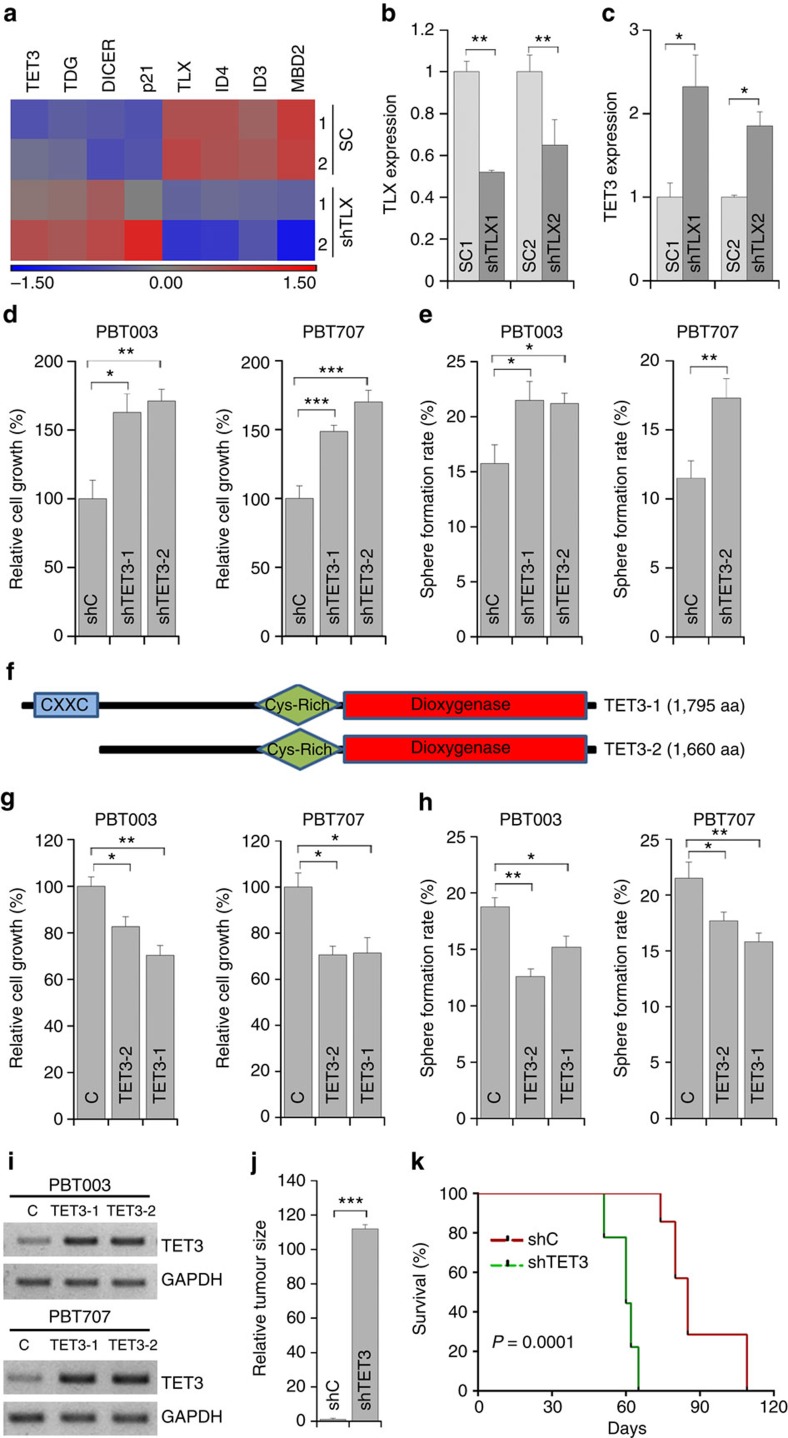
TET3 regulates GSC self-renewal and tumorigenesis. (**a**) Microarray analysis of PBT003 cells transduced with virus expressing scrambled control (SC) or TLX shRNA (shTLX). (**b**,**c**) RT–PCR analysis showing TET3 upregulation upon TLX knockdown. *N*=3, *P<0.05, **P<0.01 by Student's *t*-test. Error bars are s.d. of the mean. (**d**,**e**) Cell growth (**d**) and sphere formation (**e**) analyses of the GSC lines transduced with control RNA (shC) or TET3 shRNAs (shTET3-1, shTET3-2). (**f**) Schematics of TET3-1 and TET3-2 proteins with characteristic domains. (**g**,**h**) Cell growth (**g**) and sphere formation (**h**) analyses of GSCs transduced with control (C) or TET3-expressing lentivirus, TET3-1 or TET3-2. (**i**) RT–PCR analysis showing overexpression of TET3 in GSCs. (**j**) Relative sizes (volumes) of brain tumours derived from PBT003 cells that were transduced with control RNA (shC) or TET3 shRNA (shTET3). (**k**) Survival curves of NSG mice transplanted with PBT003 cells transduced with control RNA (shC) or TET3 shRNA (shTET3). *X* axis represents days after GSC transplantation. For **d**,**g**, *N*=4; for **e**,**h**, *N*=6. Error bars are s.e. of the mean. *P<0.05, **P<0.01, ***P<0.001 by Student's *t*-test. For **j**, *N*=3, ***P<0.001 by Student's *t*-test. Error bars are s.d. of the mean. For **k**, *N*=8, *P*<0.001 by log-rank test.

To investigate the mechanism by which TLX controls the self-renewal and tumorigenesis of GSCs, microarray analysis was performed to compare gene expression profiles in control and TLX shRNA-treated GSCs. *TLX* was identified in the downregulated gene cohort, whereas the cyclin-dependent kinase inhibitor *p21*, a known downstream target that is repressed by TLX^45^, was among the upregulated genes, confirming the effectiveness of TLX knockdown in TLX shRNA-treated cells. Several potential downstream targets that were not linked with TLX before were identified in our array analysis (Fig. 7a). Specifically, the expression of *TET3*, *TDG* and *DICER1* genes were upregulated and the expression of *ID3*, *ID4* and *MBD2* genes were downregulated in TLX-shRNA-treated PBT003 cells (Fig. 7a). The regulation of TET3 by TLX knockdown was confirmed in PBT003 cells and other GSC lines by RT–PCR (Fig. 7b,c and Supplementary Fig. 7a). Up–regulation of *TET3* upon TLX knockdown was also confirmed in PBT003-grafted brain tumours from NSG mice treated with virus expressing TLX shRNA compared with that in tumours from control mice (Supplementary Fig. 8). Of note, TET3, TDG and MBD2 are all involved in DNA methylation modification, suggesting epigenetic regulation of DNA methylation may be an important downstream event of knocking down TLX in GSCs.

Based on the negative regulation of TET3 expression by TLX, we hypothesized that TET3 could function as a tumour suppressor to control GSC growth and self-renewal. To test this hypothesis, two shRNAs were designed to knockdown TET3 in GSCs. Knockdown of TET3 was confirmed in PBT003 and PBT707 cells (Supplementary Fig. 7b). PBT003 cells expressing TET3 shRNAs showed increased cell growth compared with control RNA-treated cells (Fig. 7d). Consistent with increased cell growth, PBT003 cells with TET3 knockdown also showed increased sphere formation rate compared with control cells (Fig. 7e). The increased cell growth and sphere formation rate after knockdown of TET3 were also observed in PBT707 cells (Fig. 7d,e). Taken together, these results demonstrated that knockdown of TET3 increased GSC growth and self-renewal.

To investigate if TET3 is sufficient to regulate the growth and self-renewal of GSC, we tested the effect of TET3 overexpression in GSCs. Human *TET3* gene *TET3-1* (with the CXXC domain) or *TET3-2* (without the CXXC domain; Fig. 7f) was cloned into a lentivirus vector. Both TET3-1 and TET3-2 contain the dioxygenase domain that is present in all TET proteins. Overexpression of TET3-1 and TET3-2 was confirmed by RT–PCR in PBT003 and PBT707 cells transduced with the TET3-expressing virus (Fig. 7i and Supplementary Fig. 7c,d). Overexpressing either TET3-1 or TET3-2 reduced the growth of both PBT003 and PBT707 cells (Fig. 7g). Consistent with decreased cell growth, PBT003 and PBT707 cells overexpressing TET3-1 or TET3-2 also showed decreased sphere formation rate compared with control cells (Fig. 7h). These results together indicate that TET3 inhibits GSC growth and self-renewal.

To investigate if TET3 regulates GSC tumorigenicity, PBT003 cells were transduced with a lentiviral vector expressing a TET3 shRNA or a control shRNA. The transduced cells were then transplanted into the frontal lobe of NSG mouse brains. Tumour formation and expansion by the TET3 shRNA-treated GSCs were compared with that by control RNA-treated GSCs maintained under identical conditions. Stereological measurement of tumour volumes confirmed the development of significantly larger tumours in brains transplanted with GSCs treated with TET3 shRNA, compared with that in brains transplanted with control GSCs (Fig. 7j). Mice transplanted with TET3 shRNA-transduced PBT003 cells had significantly shorter survival compared with mice transplanted with control RNA-transduced cells (Fig. 7k). Together, these results indicate that knockdown of TET3 increases tumour progression and decreases the lifespan of GSC-grafted mice, in a manner opposite to knockdown of TLX.

### TET3 acts downstream of TLX to regulate GSC self-renewal

**Figure 8:**
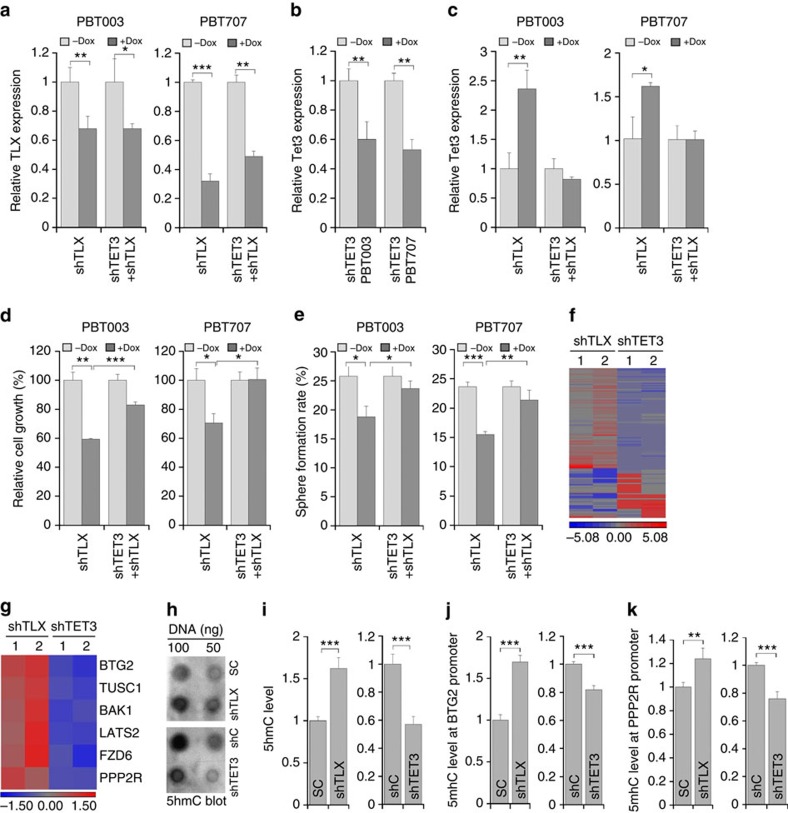
TET3 acts downstream of TLX to regulate GSC growth and self-renewal. (**a**–**c**). RT–PCR analysis of dox-induced knockdown of TLX (**a**) and TET3 (**b**,**c**) in PBT003 and PBT707 cells transduced with lentivirus expressing dox-inducible TLX shRNA (shTLX) and or with dox-inducible TET3 shRNA (shTET3 or shTET3+shTLX). *N*=3, error bars are s.d. of the mean. *P<0.05, **P<0.01, ***P<0.001 by Student's *t*-test for all the quantifications in this figure. (**d**,**e**) Cell growth (**d**) and sphere formation (**e**) analyses of PBT003 and PBT707 cells transduced with lentivirus expressing dox-inducible shTLX alone or together with dox-inducible shTET3. *N*=4 for **d** and *N*=6 for **e**, error bars are s.e. of the mean. (**f**) Heat map of differentially expressed genes in PBT003 cells transduced with virus expressing TLX shRNAs (shTLX1, shTLX2) or TET3 shRNAs (shTET3-1, shTET3-2), in microarray analysis. (**g**) Heat map of six differentially expressed genes in PBT003 cells transduced with virus expressing TLX shRNAs (shTLX1, shTLX2) or TET3 shRNAs (shTET3-1, shTET3-2), in microarray analysis. (**h**,**i**) 5hmC dot blot analysis of total 5hmC level in TLX knockdown or TET3 knockdown PBT003 cells. (**j**,**k**) Hydroxymethylated DNA immunoprecipitation (hMeDIP)-qPCR analysis of BTG2 and PPP2R1B (PPP2R) promoter in PBT003 cells transduced with virus expressing shTLX or shTET3. *N*=4, error bars are s.d. of the mean.

To test whether TET3 acts downstream of TLX to regulate GSC growth and self-renewal, an inducible system to double knock down TLX and TET3 was established. PBT003 and PBT707 cells were transduced with lentivirus that expresses doxycycline (dox)-inducible TLX shRNA together with a puromycin-resistant reporter gene. After puromycin selection, the stably transduced cells were then transduced with lentivirus expressing dox-inducible TET3 shRNA. Induced knockdown of TLX and TET3 was confirmed in dox-treated PBT003 and PBT707 cells transduced with lentivirus expressing dox-inducible TLX shRNA (Fig. 8a) or TET3 shRNA (Fig. 8b). As expected, the expression of TET3 was upregulated after dox-induced knockdown of TLX (Fig. 8c). Dox-induced TET3 knockdown reversed the expression of TET3 to control levels in cells expressing both inducible TLX shRNA and inducible TET3 shRNA (Fig. 8c).

Next we tested whether inducible TET3 knockdown could rescue the inhibitory effect of inducible TLX knockdown on GSC growth and self-renewal. After dox induction, the growth of PBT003 and PBT707 cells expressing inducible TLX shRNA was reduced when compared with non-induced cells (Fig. 8d). The decreased cell growth resulted from induced TLX knockdown was rescued substantially by induced TET3 knockdown in both PBT003 and PBT707 cells (Fig. 8d). The reduced self-renewal of PBT003 and PBT707 cells resulted from dox-induced TLX knockdown was also rescued by dox-induced TET3 knockdown (Fig. 8e). These results indicate that TET3 is a critical downstream target of TLX in regulating GSC growth and self-renewal.

The inverse correlation between TET3 and TLX expression in TLX knockdown GSCs led us to hypothesize that TLX, a known transcription factor that usually works as a transcriptional repressor^45^, could repress TET3 expression by directly binding to the promoter of the *TET3* gene. Chromatin immunoprecipitation (ChIP) analysis using a TLX-specific antibody revealed that TLX bound to the promoter and proximal intron regions of *TET3* containing the putative TLX-binding sites in both PBT003 and PBT707 cells (Supplementary Fig. 9a,b). At position 1 (P1, around the promoter region) and position 5 (P5, intron 2), the binding sites of TLX was associated with a higher level of repressive histone mark H3K9me3 than that of active histone mark H3K4me3 in both PBT003 and PBT707 cells (Supplementary Fig. 9c). These data suggest that TLX could regulate the transcription of *TET3* by directly binding to regulatory regions of the *TET3* gene.

To identify downstream targets of TET3 in GSCs, microarray analysis was performed. The gene expression profile of PBT003 cells treated with TET3 shRNA-expressing lentivirus was compared with PBT003 cells expressing control shRNA. Data sets from microarray analysis of PBT003 cells transduced with TLX shRNA or control RNA were included for comparison. Among the differentially expressed genes, an inverse correlation in gene expression was observed in TLX knockdown cells and TET3 knockdown cells (Fig. 8f), consistent with our hypothesis that TLX represses TET3 expression. Specifically, genes related to tumour-suppressive function, including *BTG2*, *TUSC1*, *BAK1*, *LATS2*, *FZD6* and *PPP2R1B*, were upregulated in TLX knockdown cells, but downregulated in TET3 knockdown cells (Fig. 8g and Supplementary Fig. 10), suggesting that the TLX-TET3 regulatory cascade could regulate the growth and self-renewal of GSCs through regulating these downstream tumour suppressors.

Because TET3 is a dioxygenase that converts 5mC to 5hmC, we next tested whether the TLX-TET3 regulatory cascade could regulate 5hmC level in GSCs. Dot blot analysis using a 5hmC-specific antibody revealed increased 5hmC level upon TLX knockdown, but decreased 5hmC level upon TET3 knockdown in PBT003 cells (Fig. 8h,i). Our DNA microarray analyses have identified several tumour suppressors as candidate downstream targets of TLX and TET3, including *BTG2* and *PPP2R1B* (Fig. 8g), which were also upregulated upon TLX knockdown in PBT003-grafted brain tumours in NSG mice (Supplementary Fig. 8). To test whether the TLX-TET3 cascade regulates 5hmC level at the promoter of these potential downstream targets, hydroxymethylated DNA immunoprecipitation-quantitative PCR (qPCR) analysis was performed. The 5hmC levels at the promoter region of *BTG2* and *PPP2R1B* genes were significantly increased in PBT003 cells transduced with TLX shRNA, compared with control cells (Fig. 8j,k), consistent with the elevated expression of these genes in TLX knockdown PBT003 cells (Supplementary Fig. 10a). In contrast, knockdown of TET3 reduced 5hmC level at the promoter region of *BTG2* and *PPP2R1B* (Fig. 8j,k), consistent with the reduced expression of these genes in TET3 knockdown PBT003 cells (Supplementary Fig. 10b). These results indicate that the TLX-TET3 regulatory cascade could regulate the expression of downstream tumour-suppressor genes by controlling the 5hmC level at their promoter regions.

## Discussion

Cancer stem cells are critical for tumour maintenance, metastasis and resistance to therapy. Therefore, targeting cancer stem cells is a priority in the development of novel cancer therapies that can completely cure and eradicate cancer by eliminating residual tumour-initiating cells. GSCs are implicated in the initiation and development of GBM, the most aggressive and invariably lethal brain tumour. This study identified the nuclear receptor TLX and TET3 regulatory axis as a target for GSCs. The results of this study demonstrated that TLX is fundamental for maintaining GSC growth, self-renewal and *in vivo* tumour formation capacity, whereas TET3 is a potential tumour suppressor that inhibits GSC growth, self-renewal and tumorigenesis. Knockdown of TLX expression using either virally expressed shRNA or nanoparticle-delivered siRNA dramatically reduced the growth and self-renewal of GSCs *in vitro* and impaired the ability of GSCs to form brain tumours *in vivo*. The finding that *in vivo* knockdown of TLX expression inhibits brain tumour development suggests that TLX is a promising target for anti-GBM therapy.

A role for TLX in gliblastoma development has been proposed in studies using mouse models^14^,^46^. A recent study by lineage tracing demonstrated that TLX regulates the self-renewal of brain tumour stem cells in mouse brains^47^. However, there is no study yet to directly investigate the role of TLX in human GSCs *in vivo*. In this study, we showed direct evidence that targeting TLX in GSCs derived from human GBM patients efficiently inhibited the growth, self-renewal and tumorigenicity of GSCs *in vitro* and *in vivo*. The inhibitory effects of growth and self-renewal by TLX suppression were seen in all GSC lines tested, including classical, mesenchymal and proneural subtypes. These results suggest a general role for TLX in maintaining human GSC self-renewal, independent of GBM subtypes. Treatment of human GSC-grafted mice with TLX siRNA dramatically reduced tumour growth and significantly prolonged survival of GSC-grafted mice. Targeting TLX *in vivo* is effective at different time points, both 1 week and 2 weeks after tumour establishment. Our study, by knocking down TLX *in vivo*, provided proof-of-concept that targeting TLX is effective to suppress the progression of human GSC-derived tumours.

Our study, by knocking down TLX expression in GSCs, demonstrates that TLX is necessary for GSC self-renewal and tumorigenesis. Because TLX is an essential regulator of neural stem cell self-renewal^11^, it is possible that neural stem cells expressing high levels of TLX that beyond certain threshold may progress into gliomas over time. However, in a study using a transgenic model of TLX overexpression in neural stem cells, we did not detect any tumour formation^48^. In a separate study using a mouse model of TLX overexpression, no tumour formation was detected in brains of TLX-overexpressing mice under normal condition either^14^. Only when combined with loss of p53 or, to a less extent, ageing, overexpression of TLX led to glioma progression over time^14^. Therefore, although our study indicates that TLX is necessary for GSC self-renewal and tumorigenesis, overexpression of TLX alone may not be sufficient for GSC tumorigenesis.

Although the importance of targeting TLX in GBM is established, the knowledge on how TLX controls the self-renewal and tumorigenesis of human GSC is limited. The cyclin-dependent kinase inhibitor p21 is a known downstream target of TLX that can control cell cycle arrest. Our microarray confirmed that *p21* is upregulated upon TLX knockdown as previously reported by us and others^45^,^47^. We did not see upregulation of reported tumour-suppressor genes, such as *CDKN2A*, *CDKN2B* and *PML*, and factors for neuronal differentiation, such as *SMARCC1* and *DLX2*, in our microarray analysis. This may be due to the difference of human samples and mouse samples used for analyses. In our study, human GSCs after TLX knockdown were investigated, whereas previous study examined the expression of genes altered by TLX knockout using RNAs isolated from tumour tissues of a mouse tumour model^47^.

In this study, we identified TET3 as a potential tumour suppressor that acts downstream of TLX to regulate GSC growth and self-renewal. TLX represents the first transcription factor that has been identified to regulate TET3 expression. TET3 is a member of the TET family proteins that are known to be epigenetic regulators that control DNA demethylation. Although growing evidence showing that epigenetic regulation plays an important role in cancer development, our knowledge on the role of TET family members, especially TET3, in tumour development is rather limited. We show here that TET3 suppresses the growth, self-renewal and tumorigenesis of GSCs downstream of TLX. To our knowledge, this is the first study to define the role of TET3 in cancer stem cells and in GBM. Decreased 5hmC level has been observed in human cancers, including malignant glioma^18^,^19^,^20^, but the underling mechanism remains to be discovered. Our finding showing that TLX represses TET3 expression in GSCs could provide a plausible explanation for the decreased 5hmC level in GBM. Furthermore, we identified tumour suppressors, including *BTG2*, *TUSC1*, *BAK1*, *LATS2*, *FZD6* and *PPP2R1B*, as common targets of TLX and TET3. These tumour suppressors could be potential targets of the TLX-TET3 regulatory axis in GSCs that are worthy of further studies. The knowledge we gained about the role of TET3 in suppressing tumour stem cell growth and self-renewal, and the potential tumour-suppressor target genes we identified for TET3 in this study will trigger further studies to delineate the function of TET3 in tumour stem cells in particular and tumorigenesis in general.

Mammalian TET3 exists in isoforms either containing the CXXC domain (TET3-1) or not containing the CXXC domain (TET3-2)^49^, with estimated transcript size of 11.6 and 10.9 kb, respectively. Of interest, the TET3-2 isoform that lack the CXXC domain is the major isoform in the brain and retina^49^,^50^. Although the TET3-1 isoform can bind to DNA using its amino-terminal CXXC domain^51^, the TET3-2 form can also bind to DNA, presumably through its interacting proteins, including the CXXC domain containing protein CXXC4 (ref. 49), and transcription factors, such as REST^50^. It is clear that the TET3-2 isoform that lacks the CXXC domain is able to induce 5hmC formation and gene expression^50^. Therefore, it is not surprising that the CXXC domain of TET3 seems dispensable for the effect on inhibition of GSC growth and self-renewal we observed in this study.

Small RNAs have gained increasing attention as candidate agents for therapies. However, the success of therapeutic application of small RNAs depends on efficient intracellular delivery. Safe and efficient small RNA delivery is in urgent need. In this study, we developed an efficient system of siRNA delivery into GSCs using polycationic PAMAM dendrimer G5. This dendrimer has been shown to compact small RNAs into nanoparticles and protect RNAs from enzymatic degradation, therefore providing an efficient delivery means for introducing small RNAs into GSCs. Moreover, coating the dendrimer-siRNA nanoparticles with the tumour-homing RGDK peptide allows tumour-specific targeting. In this study, we show that GSCs express both integrin αv and neuropilin-1. Furthermore, we demonstrated that the RGDK-coated dendrimer-TLX siRNA nanoparticles were able to deliver TLX siRNAs into GSCs efficiently and exert potent gene knockdown and growth inhibitory effect. When delivered *in vivo*, the RGDK-coated dendrimer-TLX siRNA nanocomplex inhibited the growth of human GSC-initiated tumours. Moreover, treatment of the RGDK-coated G5-TLX siRNA complex significantly extended the lifespan of tumour-bearing mice, increasing the median survival from 48 days after the first treatment to 53 days, corresponding to about 7-month prolonged survival in patients. Similarly, mild but significant increase of survival has also been observed by targeting other important GBM targets^52^,^53^.

A potential clinical significance of this finding is derived from the ability of knocking down TLX in GSCs via lentivirus-delivered shRNA or nanoparticle-delivered siRNA to compromise the self-renewal and tumour formation potential of GSCs *in vivo*. Our studies demonstrated that TLX knockdown inhibited tumour initiation and progression from human GSCs in a xenografted tumour model and increased the survival of grafted animals substantially. GBM is highly aggressive brain tumour with a short life expectancy of a little over a year after diagnosis, and patient survival is only marginally increased by current therapies. The small RNA approach to knock down TLX in GSCs has the potential to help improve the outcome and survival of GBM patients.

## Methods

### Cell culture

Sphere cultures of GSCs were established from freshly dissociated surgical specimens as described^36^. Patients were newly diagnosed as grade IV GBM multiforme based on World Health Organization-established guidelines. All patient tissues were obtained in accordance with the City of Hope Institutional Review Board-approved protocols. The study uses completely anonymized specimens. No informed consent is involved. The GSCs were maintained in DMEM-F12 medium (Omega Scientific) supplemented with 1X B27 (Invitrogen), 5 μg ml^−1^ heparin (Sigma), 2 mM L-glutamine (Media Tech), 27.4 mM HEPES (Fisher), 20 ng ml^−1^ EGF (PeproTech) and 20 ng ml^−1^ FGF (PeproTech) with growth factors replenished twice a week. GSCs were treated with accutase (Innovative Cell Technologies) for cell dissociation and induced into differentiation using 0.5% fetal bovine serum and 1 μM all-*trans* retinoic acid. All the cultures used in this study were confirmed for the lack of mycoplasma contamination using MycoAlert PLUS Mycoplasma Detection Kit (Lonza).

### Plasmid DNA and viral preparation and transduction

shRNAs or the scrambled control RNAs were cloned into lentiviral pHIV7-GFP or pHIV-TetR-GFP vector. The sequences for shRNAs include TLX shRNA-1 (5′-GCCGCCATTGCAGCCCTTCAA-3′) and TLX shRNA-1 scrambled control (5′-CAGTCCATCAGACCCTCGCTG-3′), TLX shRNA-2 (5′-GGAAGTCAACATGAACAAAGA-3′) and TLX shRNA-2 scrambled control (5′-ACTCAAAAGGAAGTGACAAGA-3′), shRNA control for TET3 (5′-GTTCAGATGTGCGGCGAGT-3′), shTET3-1 (5′-CCGAAGCTGTGTCCTCTTA-3′) and shTET3-2 (5′-GGAGTCACCTCTTAAGTAC-3′)^54^. Lentiviruses were produced using 293T cells. To transduce GSCs, spheres were dissociated and incubated with lentivirus and 4 μg ml^−1^ polybrene for 24 h. Human TET3-expressing vector EX-H2292-M11 was purchased from GeneCopoeia and hTET3 sequences were subcloned into CSC lentiviral vector to get TET3-2-expressing vector. Human TET3 CXXC domain sequences were amplified from human neural stem cell cDNAs and subcloned into TET3-2-expressing vector to get TET3-1-expressing vector that expresses full length of *TET3* gene.

### RT-PCR

Total RNAs were isolated with Trizol reagent (Invitrogen) or RNeasy Mini Kit (Qiagen). Reverse transcription (RT) was performed using the Tetro cDNA synthesis Kit (BioLINE). RT–PCR reactions were performed using SYBR Green Master Mix (Thermo Scientific) on Step One Plus Real-Time PCR instrument (Applied Biosystems). The primers for RT–PCR are listed in Supplementary Table 1. Actin or glyceraldehyde-3-phosphate dehydrogenase was used as the reference gene for normalization. The ΔΔCt method was used for quantification analysis.

### Immunostaining

For immunofluorescence, cells were fixed with 4% paraformaldehyde and permeabilized in 0.3% Triton X-100. Antibodies included rabbit anti-TLX (1:1,000; Shi lab)^45^, mouse anti-nestin (1:2,000; BD Pharmingen; Catalogue # 611659)^55^, rabbit anti-integrin αv (1:500; Chemicon; Catalogue # AB1923)^43^, mouse anti-neuropilin-1 (1:11; Miltenyi Biotec; Catalogue # 130-090-693)^43^, mouse anti-GFAP (1:1,000; Sigma; Catalogue # G3893) and rabbit anti-Tuj1 (1:6,000; Covance; Catalogue # PRB-435P)^56^.

### Animals

All animal-related work was performed under the IACUC protocol 05050 approved by the City of Hope Institutional Animal Care and Use Committee. Both male and female NSG mice (the Jackson Laboratory) at 6–8 weeks old were used at age- and gender-matched manner. The sample size was determined based on using *t*-test for two-group independent samples to reach power of 0.8 and the significance level of 0.05. *P*<0.05 was considered statistically significant. When monitoring tumour growth, investigators were blind to the group allocation during the bioluminescence xenogen imaging and aware of group allocation when assessing the outcome.

### Viral transduction followed by transplantation

GSCs were transduced with control RNA or relevant shRNA expressing lentivirus. Two days after virus transduction, 5 × 10^4^ cells were transplanted into the frontal lobes of brains of 6- to 8-week-old NSG mice by stereotaxic intracranial injection. Briefly, 2 μl dissociated cells in PBS were injected into the following site (AP=+0.6 mm, ML=+1.6 mm and DV=−2.6 mm) with a rate of 1 μl min^−1^. The same coordinates were used for all intracranial injections in this study. Mouse brains were harvested when severely sick mouse was found in treated or control group. H&E staining was performed on 20 μm coronal sections of frozen brain samples, followed by tumour size analysis. The tumour volume was measured by multiplying the area of the tumour tissues (quantified by Image J) by the thickness of the sections, then multiplying by the number of the sections that contain the tumour tissues. In a separate set of experiment, the survival of grafted mice was recorded and analysed.

### Intracranial viral transduction

PBT003 cells (2 × 10^5^) transduced with luciferase-expressing lentivirus were intracranially transplanted into the frontal lobe of 6- to 8-week-old NSG mice. One week or two weeks later, mice were randomly grouped and treated with scrambled control RNA or TLX shRNA-expressing lentivirus by intratumoral injection. Tumour growth was monitored by bioluminescence xenogen imaging. The bioluminescence intensity was quantified. Six weeks after virus treatment, mouse brains were collected and H&E staining was performed on brain sections. In a separate set of experiment, the survival of mice after virus treatment was recorded and analysed.

### Dendrimer-based siRNA delivery *in vitro*

The G5 dendrimer was synthesized starting with the triethanolamine core and following the iterative Micheal addition and amidation as previously described^38^. The RGDK peptide was synthesized as an oligopeptide with the sequence of E_16_G_6_RGDK^40^ and purchased from GL Biochem Ltd. The TLX siRNA has the sequence of sense: 5′-CCGCCAUUGCAGCCCUUCAAGAUdGdA-3′, antisense: 5′-UCAUCUUGAAGGGCUGCAAUGGCGGGG-3′. The control siRNA has the sequence of sense: 5′-CAUCCAUCAGACCCUCGCUGGAUdGdA-3′, antisense: 5′-UCAUCCAGCGAGGGUCUGAUGGAUGGG-3′. To form RGDK-coated G5 dendrimer-siRNA complexes, G5 was first mixed with siRNAs at N/P ([total terminal amines in G5]/[phosphates in siRNA]) ratio of 5 and kept at 37 °C for 30 min. Then RGDK peptide was added to the G5-siRNA complex at G5/RGDK molar ratio of 0.426 and incubated at 37 °C for another 10 min. The G5-siRNA or RGDK-G5-siRNA complexes were then added to GSCs. PBT003 cells (2 × 10^5^) were treated with Cy3-siRNA alone (50 nM), G5 dendrimer-siRNA or RGDK-coated G5 dendrimer-siRNA (N/P ratio of 5; G5/RGDK ratio of 0.426). Two days after, cellular uptake of Cy3-labelled siRNA delivered by siRNA alone, G5 dendrimers or RGDK-coated G5 dendrimers was monitored by fluorescence microscopy and flow cytometry. For knocking down of TLX, PBT003 cells were treated with the G5 dendrimer-TLX siRNA complex or G5-control siRNA complex with or without RGDK coating, TLX expression was analysed by RT–PCR after 2 days of dendrimer-TLX siRNA treatment.

### Intracranial delivery of dendrimer-siRNA nanoparticles

PBT003 cells (2 × 10^5^) transduced with luciferase-expressing lentivirus were intracranially transplanted into the frontal lobe of 6- to 8-week-old NSG mice. One week after transplant, tumours were detected by bioluminescence imaging and mice were treated with RGDK-coated G5 dendrimer-TLX siRNA complex or RGDK-coated G5 dendrimer-SC complex (2.5 nmole siRNA per mouse with N/P ratio of 5, G5/RGDK ratio of 0.426) by intratumoral injection once a week for 6 weeks. Tumour growth was monitored by bioluminescence imaging once a week for 7 weeks. The bioluminescence intensity, mouse body weight and mouse survival were analysed.

### Microarray analysis and GBM subtype determination

For GBM subtype characterization, total RNAs were extracted from ten lines of GSCs using RNeasy Mini Kit (Qiagen). Microarray analysis was performed using GeneChip Human Genome U133A 2.0 Array (Affymetrix). Microarray labelling, hybridization and quality control measurements were performed in the Integrative Genomics Core of City of Hope. The microarray expression data of the ten GSC samples were pooled together with published microarray expression data of 173 TCGA samples^37^. After batch removal, principle component analysis (PCA) was performed on the pooled data set using Partek Genomics Suite software, version 6.6 (2014 Partek Inc). 747 relevant genes were used for PCA according to the data filtering approach as described^37^. Centroids of the four GBM subtype clusters were defined by PCA and the Euclidean distance of each GSC sample to the centroids were calculated. Samples were classified to the GBM subtype that has the least Euclidean distance value.

To identify TLX or TET3 downstream target genes, PBT003 cells were transduced with scrambled control RNA or TLX shRNA-expressing lentivirus (for shTLX microarray), or control RNA or TET3 shRNA-expressing lentivirus (for shTET3 microarray). Three days after virus transduction, total RNA was extracted using RNeasy Mini Kit (Qiagen). Microarray analysis was performed using GeneChip PrimeView Human Gene Expression Array (Affymetrix). Microarray labelling, hybridization and quality control measurements were performed in the Integrative Genomics Core of City of Hope. Microarray analysis was performed using Partek Genomics Suite (Partek, Inc.). Expression values were robust multi-array average (RMA) normalized^57^, and fold-change values were calculated using least-squares mean between samples. Genes were defined as differentially expressed if they showed an absolute value of fold-change larger than 1.5. Heat maps to visualize differentially expressed genes were produced in Partek using Euclidian distance for hierarchical clustering of standardized expression values. Microarry a data have been deposited to NCBI's GEO under accession number GSE75945.

### Cell growth and sphere formation analysis

PBT cells were transduced with relevant shRNA or specific gene-expressing lentivirus. For growth analysis, the transduced cells were cultured for 4 to 10 days in 24-well plates, and cell numbers were counted using a haemocytometer every 2 or 3 days. For sphere formation assay, the transduced cells were seeded at 100 cells per well in 48-well plates and cultured for 2–3 weeks followed by analysis of sphere number under microscope. The sphere formation rate was defined as the percentage of sphere-forming cells out of the 100 starting cells.

### *In vitro* limiting dilution assay

PBT cells transduced with control RNA or relevant shRNA expressing lentivirus were seeded at 1, 5, 10, 20, 50 and 100 cells per well into a 96-well plate. Ten days after seeding, the number of neurospheres in each well was counted. Extreme limiting dilution analysis was performed as described^52^,^58^ using software available at http://bioinf.wehi.edu.au/software/elda.

### CellTiter-Glo luminescent assay

PBT003 cells were treated with G5 dendrimers or RGDK-coated G5 dendrimers complexed with 100 nM scrambled control RNA or TLX siRNA at N/P ratio of five. Three days after treatment, cells were seeded at 5,000 cells per well in a 96-well plate. After another 3 days, cells were subjected to CellTiter-Glo luminescent assay (Promega). The luminescent intensity is an indication of relative cell number.

### Dox inducible knockdown

PBT003 or PBT707 cells were transduced with lentivirus expressing dox-inducible TLX shRNA, with or without lentivirus expressing dox-inducible TET3 shRNA. PBT003 cells (1 × 10^5^) or PBT707 cells (5 × 10^4^) were induced with dox (5 μg ml^−1^ for PBT003, 2 μg ml^−1^ for PBT707) or without dox every other day for 6 days (for PBT003) or 4 days (for PBT707), followed by cell counting using a haemocytometer. PBT003 or PBT707 cells without viral transduction were used as controls to test toxicity from dox. For gene knockdown effect, total RNA was extracted 4 days after dox induction, followed by RT–PCR.

### *In vivo* gene knockdown

PBT003 cells (2 × 10^5^) were intracranially transplanted into the frontal lobes of 6- to 8-week-old NSG mice. One week later, the transplanted mice were treated with scrambled control RNA or TLX shRNA-expressing lentivirus by intratumoral injection. Three days after viral transduction, total RNA was extracted from tumour tissues and RT–PCR was performed using human gene-specific primers to determine *in vivo* TLX knockdown and expression of downstream target genes *TET3*, *BTG2* and *PPP2R1B*.

### ChIP assay

ChIP assay was performed following our previously published procedure^45^. Briefly, 5 million PBT003 or PBT707 cells and 5 μg TLX antibody^45^, 0.5 μg trimethyl H3K4 antibody (HeK4me3, Cell Signaling; Catalogue # 9727) or 0.5 μg trimethyl H3K9 antibody (H3K9me3, Abcam; Catalogue # Ab8898-25) were used for each immunoprecipitation assay. The precipitation was performed using magnetic beads conjugated protein G (Thermo Fisher). Primers used are listed in Supplementary Table 1.

### 5hmC dot blot

PBT003 cells were transduced with relevant shRNA-expressing lentivirus. Three days after, genomic DNA was extracted (QIAamp DNA Mini Kit) and subjected to dot blot analysis using an antibody specific for 5hmC (1:5,000; Active Motif; Catalogue # 39770)^51^.

### Hydroxymethylated DNA immunoprecipitation-qPCR

PBT003 cells were transduced with relevant shRNA-expressing lentivirus. Three days after, hydroxymethylated DNA immunoprecipitation assay was performed using 5 million cells and 1 μl rabbit anti-5hmC antibody (Active Motif; Catalogue # 39770)^51^ for each reaction. The immunoprecipitation was carried out using magnetic beads conjugated protein G (Thermo Fisher). Primers used for BTG and PPP2R1B RT-PCR are listed in Supplementary Table 1.

## Additional information

**Accession codes:** The microarry data have been deposited in the GEO database under accession code GSE75945.

**How to cite this article:** Cui, Q. *et al.* Downregulation of TLX induces TET3 expression and inhibits glioblastoma stem cell self-renewal and tumorigenesis. *Nat. Commun.* 7:10637 doi: 10.1038/ncomms10637 (2016).

## Supplementary Material

Supplementary Information Supplementary Figures 1-10, Supplementary Table and Supplementary Methods
