# Supplementary material for: Downregulation of TLX induces TET3 expression and inhibits glioblastoma stem cell self-renewal and tumorigenesis
Source: Nat Commun. 2016 Feb 3;7:10637. doi: 10.1038/ncomms10637 (PMC4742843; doi:10.1038/ncomms10637)
Supplement: Supplementary Information — Supplementary Figures 1-10, Supplementary Table and Supplementary Methods [file ncomms10637-s1.pdf]

Supplementary Figures

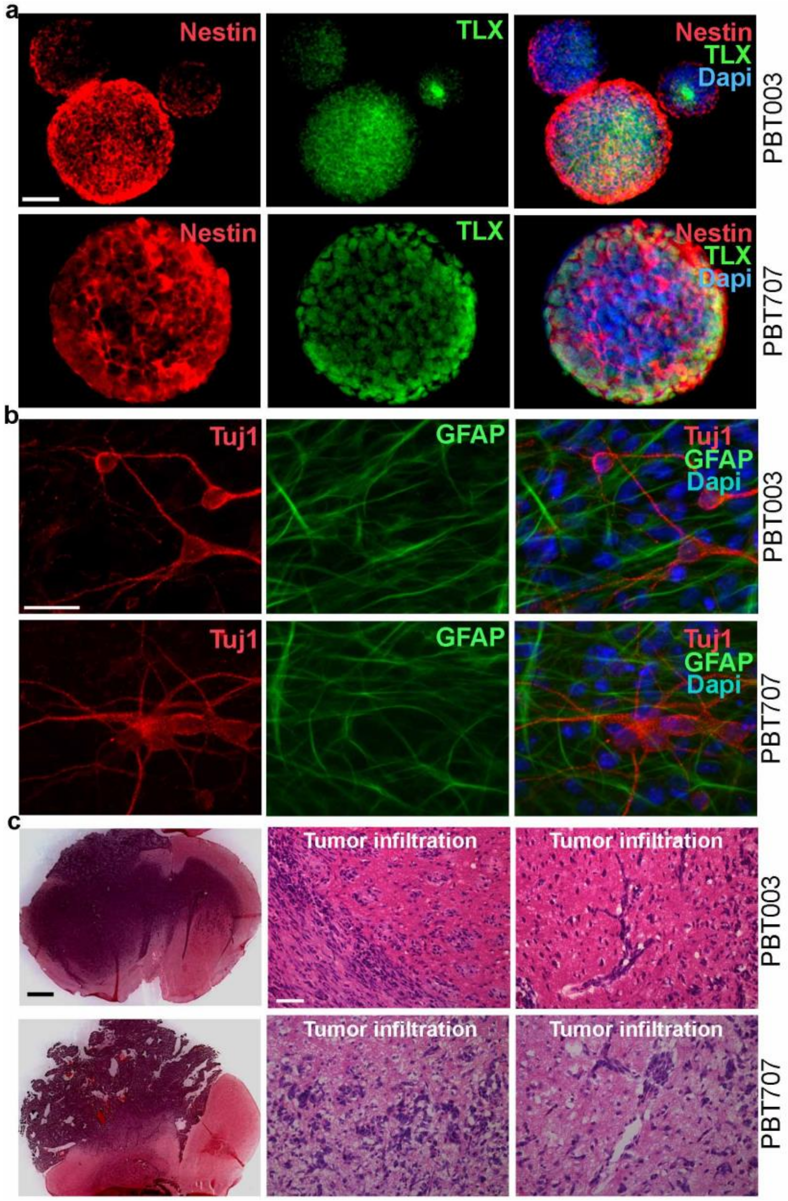

Supplementary Fig. 1 Characterization of GSCs. a. Immunostaining of primary GSC spheres from GSC lines. Nestin (neural progenitor marker, red), TLX (green). Merged images of nestin, TLX and DAPI staining (blue) are shown on the right. Scale bar: 20 µm. **b.** Multipotency of the GSCs. When induced to differentiate, the GSCs gave rise to both Tuj1-positive neurons (red) and GFAP-positive astrocytes (green). A merged image of Tuj1, GFAP, and DAPI staining (blue) is shown on the right. Scale bar: 25 µm. **c.** Tumor formation by GSCs. When transplanted into the brains of immunodeficient NSG mice, GSC lines (PBT003, PBT707) formed brain tumors with typical infiltrative characteristics of glioblastoma, as revealed by H&E staining. Scale bar: 1mm (left); 50 µm (right).

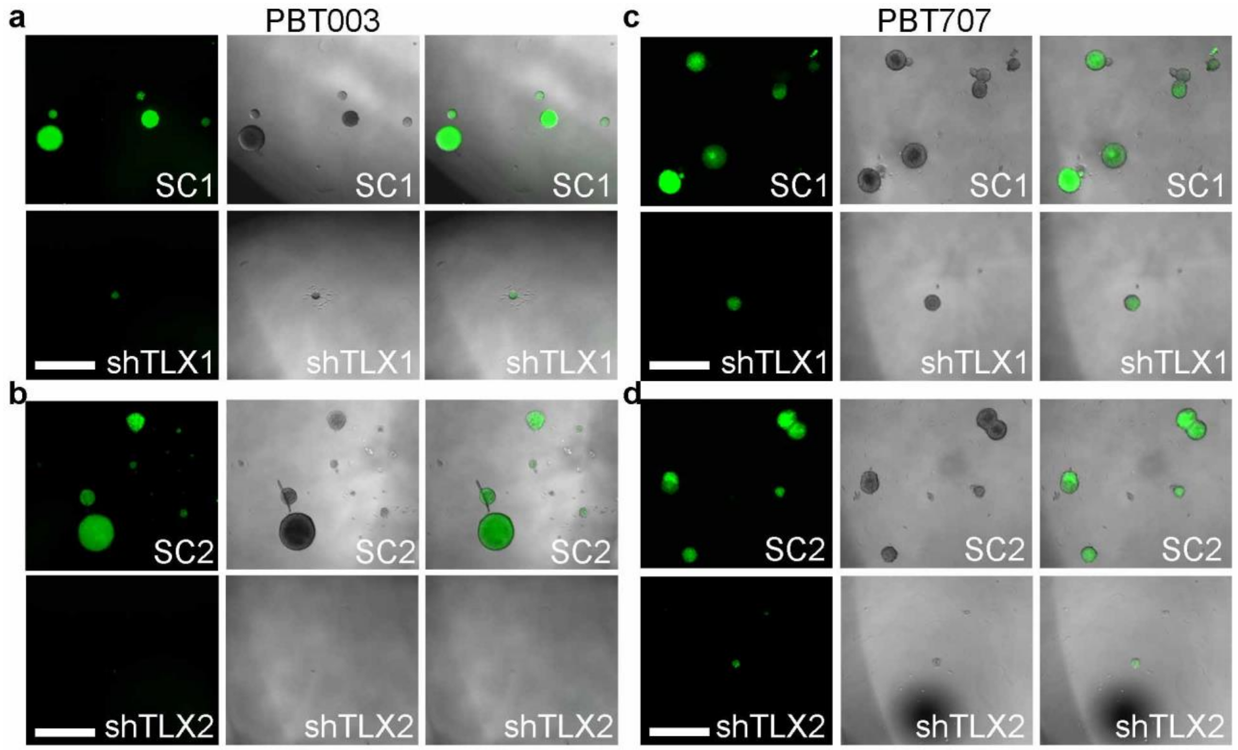

Supplementary Fig. 2 Knockdown of TLX reduces GSC self-renewal. a, b. Clonal analysis of PBT003 cells transduced with a GFP reporter and scrambled control RNAs (SC1, SC2) or TLX shRNAs (shTLX1, shTLX2). GFP fluorescence images are shown on the left, phase contrast images are shown in the center, and merged images are shown on the right. **c, d.** Clonal analysis of PBT707 cells transduced with a GFP reporter and scrambled control RNAs (SC1, SC2) or TLX shRNAs (shTLX1, shTLX2). Assays were repeated three times. Scale bar: 200 µm.

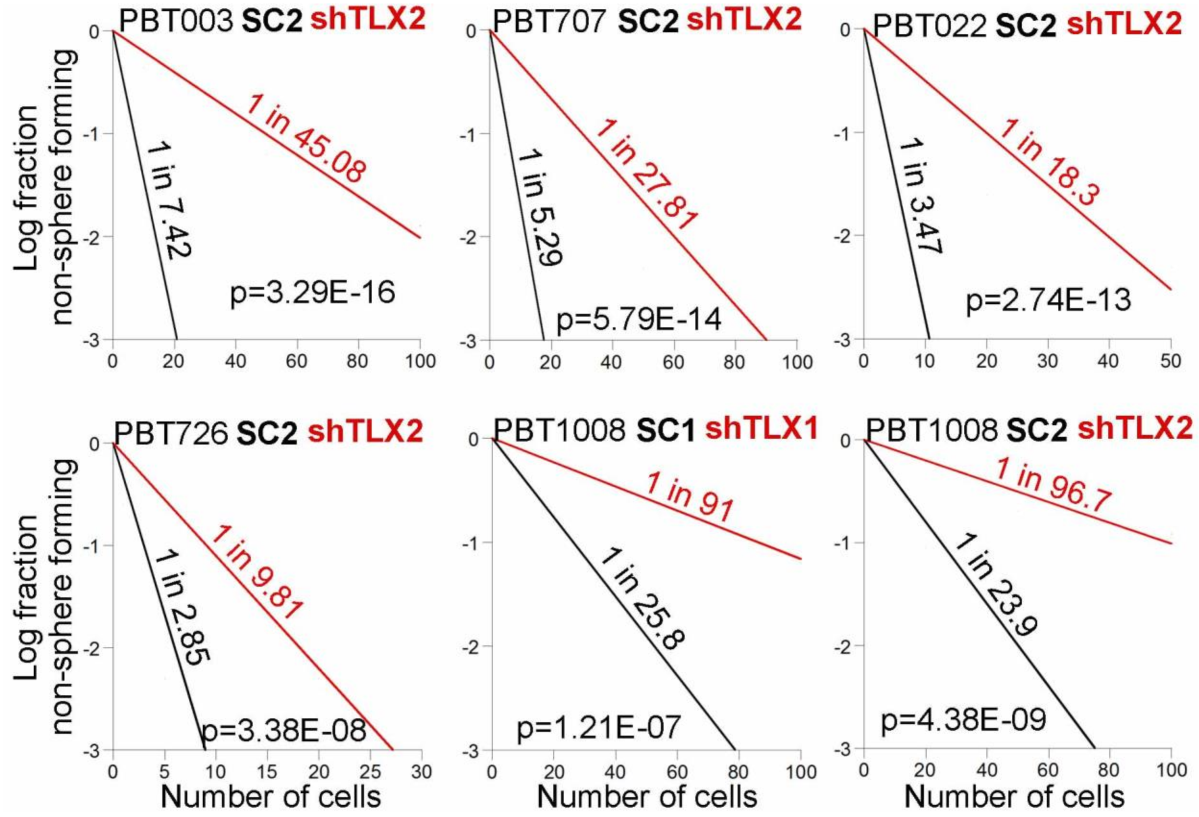

Supplementary Fig. 3 Limiting dilution assay of GSCs. Limiting dilution assay (LDA) analysis of GSCs transduced with control RNAs (SC) or TLX shRNAs (shTLX), N=20. p-value defined by one-way ANOVA test is indicated in each panel of the figure.

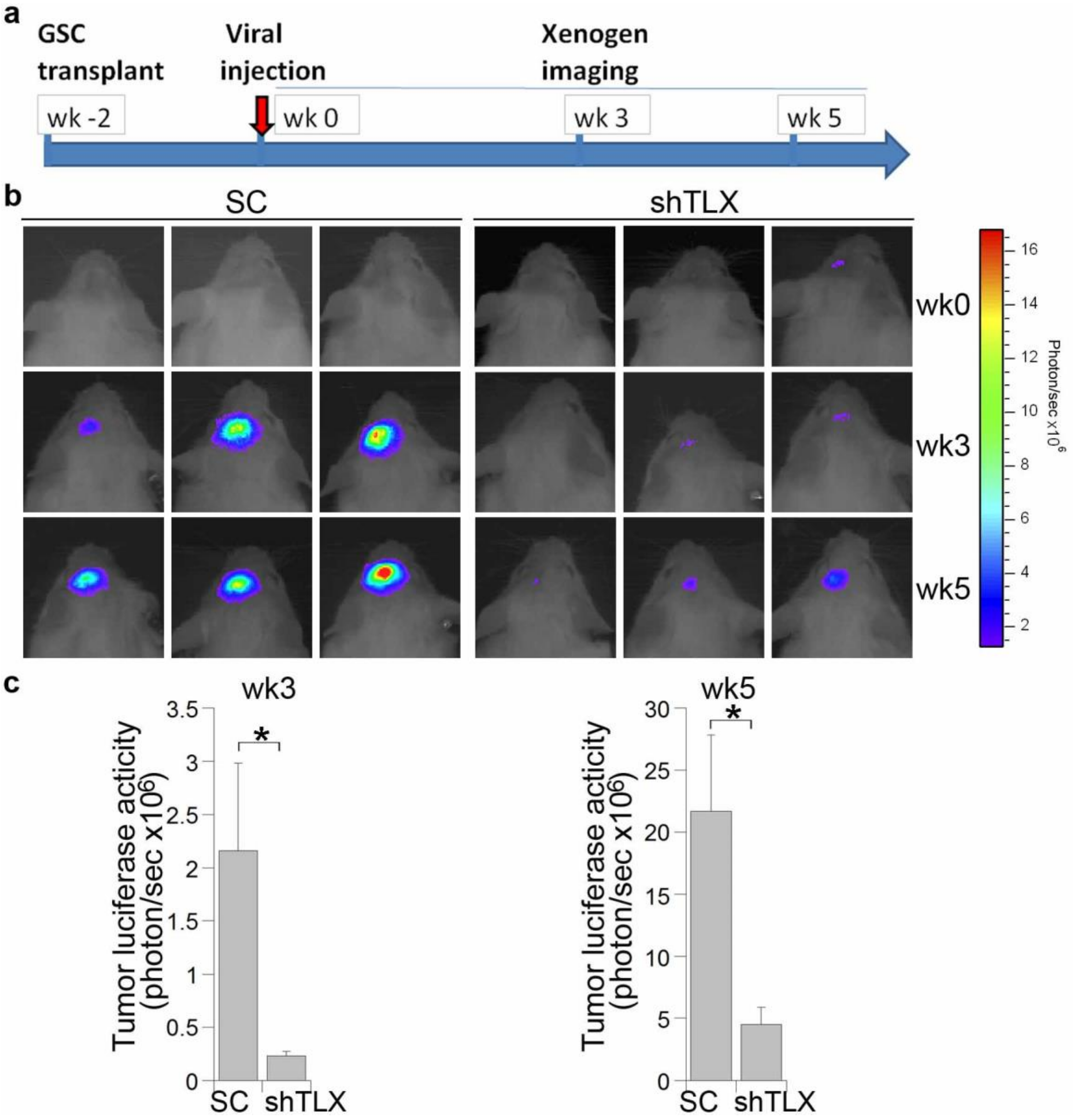

Supplementary Fig. 4 Knockdown of TLX suppresses tumor progression. a. Schematic of the experimental design, including GSC transplantation, viral treatment and xenogen imaging of xenografted tumors. **b.** Xenogen images of brain tumors in NSG mice treated with virus expressing scrambled control (SC) or TLX shRNA (shTLX). The scale for bioluminescence intensity is shown on the right. **c.** Quantification of the bioluminescence intensity of tumors treated with scrambled control (SC) or TLX shRNA (shTLX) in the brains of engrafted NSG mice. N=3, error bars are s.e. of the mean. *$p<0.05$ by Student's t-test.

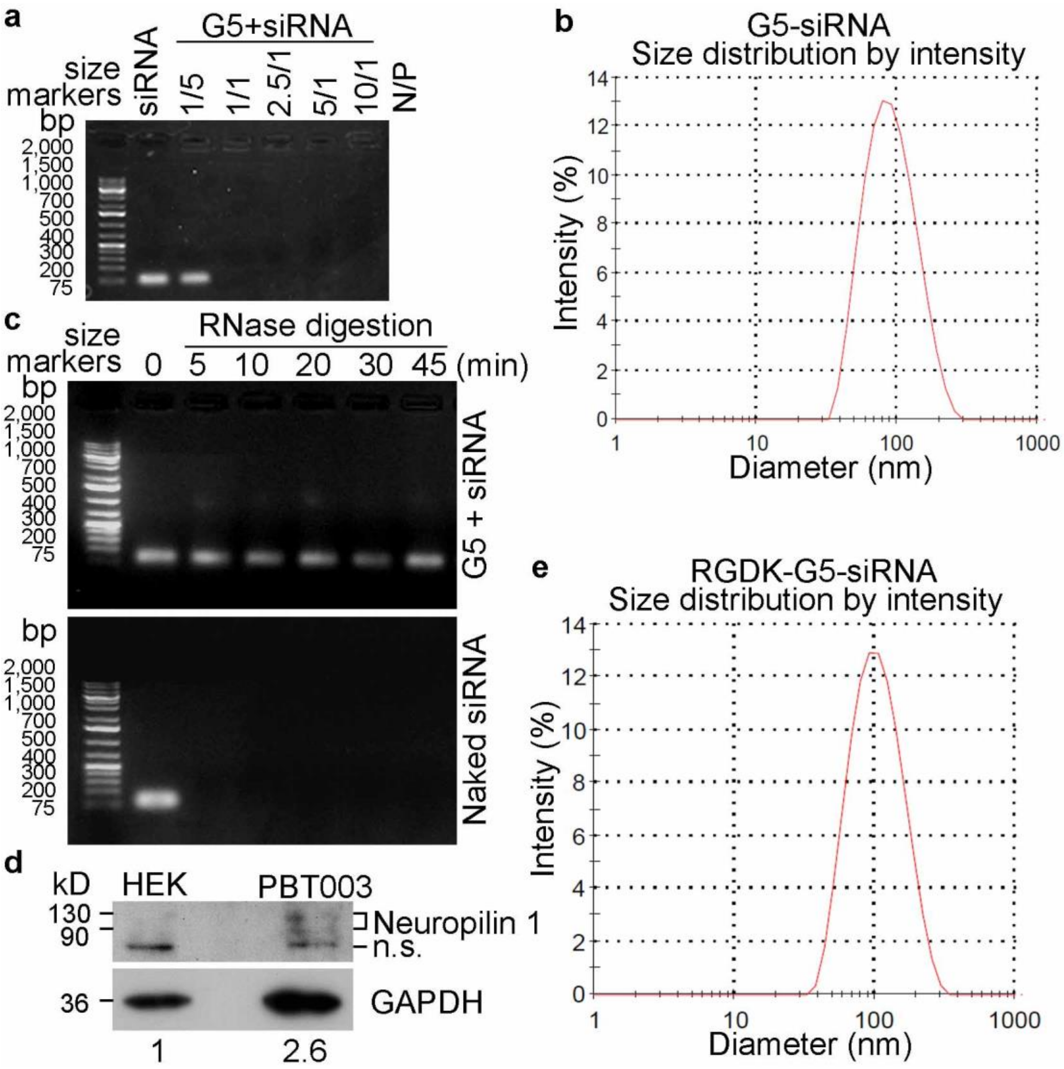

Supplementary Fig. 5 Characterization of the TLX siRNA nanocomplex. a. The binding ability of G5 with TLX siRNA at N/P ratios ranging from 1 to 10 tested using agarose gel electrophoresis. Naked TLX siRNA was used as a control. Size markers are included. **b.** Size distribution of the G5-TLX siRNA complexes at N/P ratio of 5 measured by dynamic light scattering (DLS). **c.** RNase digestion assay. Naked TLX siRNA and the G5-TLX siRNA complexes at N/P ratio of 5 were incubated with 0.01 µg per µl RNase A at 37°C for 0-45 min, followed by agarose gel electrophoresis analysis. Size markers are included. **d.** Western blot analysis of neuropilin 1 expression in PBT003 cells. HEK293T cells were included as a control. ns: non-specific signal. The size of the proteins is indicated on the left side of the images. The relative intensity of neuropilin 1 after normalization to GAPDH is shown underneath the images. **e.** Size distribution of the RGDK-G5-TLX siRNA nanoparticles at N/P ratio of 5 determined by DLS.

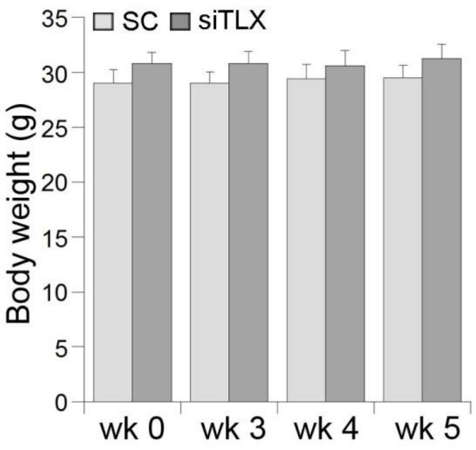

Supplementary Fig. 6 Weight analysis of TLX siRNA nanocomplex-treated mice. Measurement of mouse body weight before and after treatment of tumor bearing NSG mice by RGDK-G5-TLX siRNA complex or RGDK-G5-SC RNA complex. N=7, error bars are s.d. of the mean.

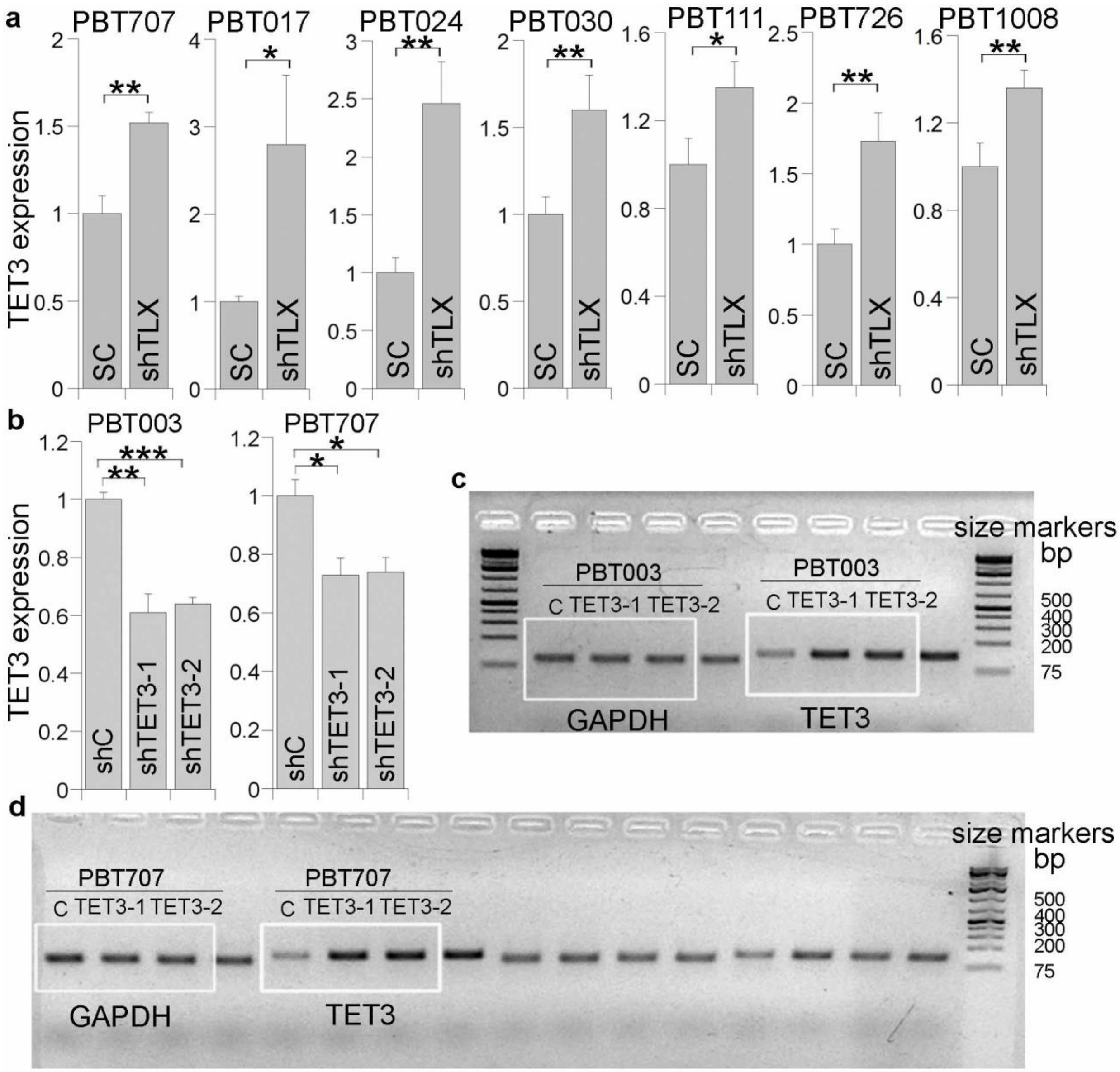

Supplementary Fig. 7 Up-regulation of TET3 in TLX knockdown GSCs. a, b. RT-PCR showing TET3 up-regulation upon TLX knockdown (**a**) and TET3 down-regulation upon TET3 knockdown (b) in GSCs. SC is the control RNA for shTLX, and shC is the control RNA for shTET3-1 and shTET3-2. N=3, *p<0.05, **p<0.01, ***p<0.001 by Student's t-test, error bars are s.d. of the mean**. c, d.** Full gels for images in Fig. 7i. Size markers are included.

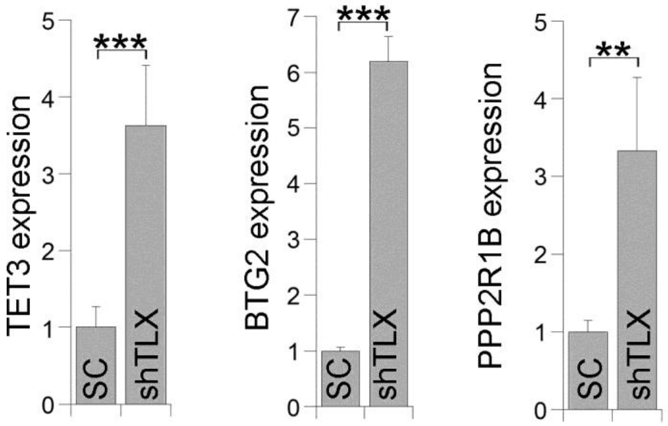

Supplementary Fig. 8 Regulation of TET3 and targets in TLX knockdown GSCs *in vivo*. RT-PCR analysis of TET3, BTG2, PPP2R1B in PBT003-grafted brain tumors treated with scrambled control (SC) or TLX shRNA (shTLX) in NSG mice. N=3, error bars are s.e. of the mean. **p<0.01, ***p<0.001 by Student's t-test.

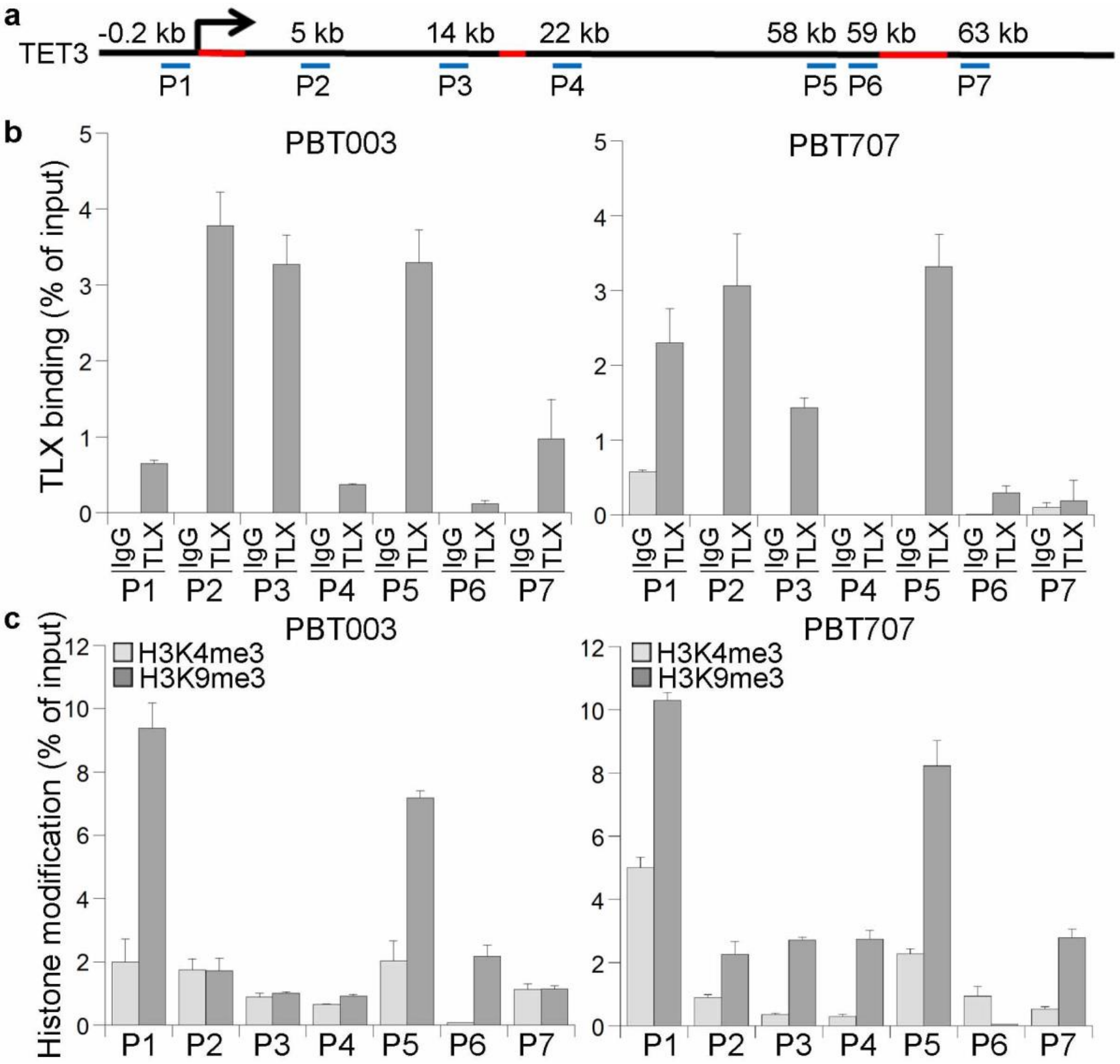

Supplementary Fig. 9 TLX binds to TET3 promoter and proximal introns in GSCs. a. A schematic of TET3 promoter and proximal intron regions. The location of primers used for mapping TLX binding and histone modifications on TET3 is indicated in blue lines. Red lines represent exons, while black lines represent regions outside of exons. P1 to P7: position 1 to position 7. **b.** ChIP assay showing TLX binding to TET3 promoter and proximal introns in PBT003 and PBT707 cells. **c.** ChIP assay showing H3K4me3 and H3K9me3 histone modifications on TET3 promoter and proximal introns in PBT003 and PBT707 cells. N=4, error bars are s.d. of the mean.

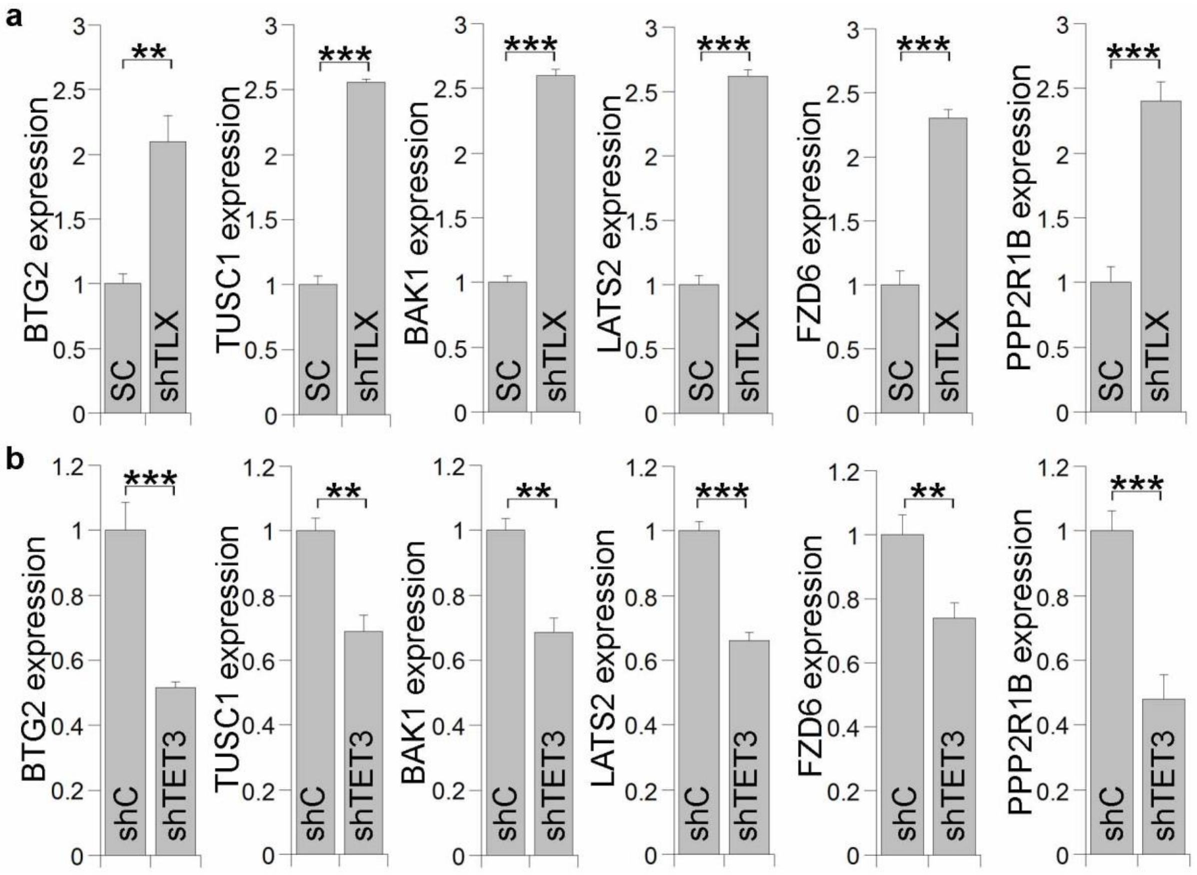

Supplementary Fig. 10 Knockdown of TET3 rescues the growth and self-renewal defects in TLX knockdown GSCs. a, b. RT-PCR analysis confirming altered expression of six tumor suppressor genes after TLX (**a**) or TET3 (**b**) knockdown in PBT003 cells. N=3, error bars are s.d. of the mean. *p<0.05, **p<0.01, ***p<0.001 by Student's t-test.

Supplementary Table 1 Primer sequences for RT-PCR and ChIP

Gene	Strand	Sequence	Assay
GAPDH	Forward	5′- CCT GTT CGA CAG TCA GCC G -3′	RT-PCR
	Reverse	5′- CGA CCA AAT CCG TTG ACT CC -3′	
Actin	Forward	5′- CCG CAA AGA CCT GTA CGC CAA C -3′	RT-PCR
	Reverse	5′- CCA GGG CAG TGA TCT CCT TCT G -3′	
TLX	Forward	5′-CTA AGA GTG TGC CAG CCT TC -3′	RT-PCR
	Reverse	5′-TGT TAG CAT CAA CCG GAA TGG -3′	
TET3	Forward	5′- CAG CAG CCG AGA AGA AGA AG -3′	RT-PCR
	Reverse	5′-GGA CAA TCC ACC CTT CAG AG -3′	
BTG2	Forward	5′- CTC CAT CTG CGT CTT GTA CGA -3′	RT-PCR
	Reverse	5′- AGA CTG CCA TCA CGT AGT TCT -3′	
TUSC1	Forward	5′- TGA AGA GGC CAG CAC GAA CC-3′	RT-PCR
	Reverse	5′- AGT CGG GTT CCT GTA GAG GC -3′	
BAK1	Forward	5′- GCT CCC AAC CCA TTC ACT AC -3′	RT-PCR
	Reverse	5′- TCC CTA CTC CTT TTC CCT GA -3′	
LATS2	Forward	5′- GTG TCT AAC TGT CGG TGT GG -3′	RT-PCR
	Reverse	5′- TCA CTC CAA CAC TCC ACC AG -3′	
FZD6	Forward	5′- CGA TAG CAC AGC CTG CAA TA -3′	RT-PCR
	Reverse	5′- ACG GTG CAA GCC TTA TTT TG -3′	
PPP2R1B	Forward	5′- TTC CAC TGT TCA CTA GTC -3′	RT-PCR
	Reverse	5′- CCA AAG TCT CAA GGT CAT C -3′	
hTLX	Forward	5′- GAC AAC TCC GGT TAG ATG CTA C -3′	RT-PCR
	Reverse	5′- GAG CCT CAT CTT GAA GGG CTG -3′	
hTET3	Forward	5′- CAG CAG CCG AGA AGA AGA AG -3′	RT-PCR
	Reverse	5′- GGC CTG GGT CCG ACG TAA TG -3′	
hBTG2	Forward	5′- CCT ATG AGG TGT CCT ACC GC -3′	RT-PCR
	Reverse	5′- CTC CGG CCC AGC AGC ACT T -3′	
TET3-P1	Forward	5′- TTG AGA AGA GGC ATC CAT CC -3′	ChIP
	Reverse	5′- AGA ACC ACA GTC GTT TCC TG -3′	
TET3-P2	Forward	5′- TGT AAT CCC AGC TCC TGA GG -3′	ChIP
	Reverse	5′- GGT TGA CAG ACT GAA CAG GG -3′	
TET3-P3	Forward	5′- ATT CTA GCC CAC CAC TCA CC -3′	ChIP
	Reverse	5′- TGT GCC AAC CAT GTT GTA GG -3′	
TET3-P4	Forward	5′- TGG CTC AGA GAA CCT CAA GG -3′	ChIP
	Reverse	5′- ACT GCC CTC CTC TGT CAT TG -3′	
TET3-P5	Forward	5′- CCT GTC GCA AAG TCA GAA TC -3′	ChIP
	Reverse	5′- TGC CCT TGT TCT CAG GAT AC -3′	
TET3-P6	Forward	5′- GTG TGT ACA CAC AGG CTT GG -3′	ChIP
	Reverse	5′- TCA CAC AAA TGA GGC TCT CC -3′	
TET3-P7	Forward	5′- GAA GAC CAG GTC AGG GTC TG -3′	ChIP
	Reverse	5′- AAG GCA AGG CTT AGA AGT GG -3′	
BTG2	Forward	5′-ACC TCC CTG GAC CTC CTG AA-3′	hMeDIP-qPCR
	Reverse	5′-TCA GTG AGA GGT CTC GGG TG-3′	
PPP2R1B	Forward	5′-CAA CGA GCT GGA TGA ATC CC-3′	hMeDIP-qPCR
	Reverse	5′-TTA AGG CTC CCT TCT GAC CC-3′	

Supplementary Methods

Agarose gel analysis of the G5-siRNA complexes

Dendrimer G5 was diluted to an appropriate concentration in 50 mM Tris-HCl buffer (pH 7.4) and stored at 4°C. The TLX siRNA was diluted in H_2O. The G5 and TLX siRNA were mixed at indicated N/P ratios and incubated at 37°C for 30 min. The final concentration of siRNA was adjusted to 25 ng per µl. Four µl of the G5-siRNA complexes were analyzed by electrophoretic mobility shift assays using 1.2% agarose gel and standard TAE buffer. The siRNA bands were stained by ethidium bromide and detected by a Kodak EDAS 290 camera.

The G5-siRNA complex protects siRNA from RNase digestion

TLX siRNA (1.2 µg) and G5 at N/P ratio of 5 were kept at 37°C for 30 min. Then the complexes were incubated in the presence of 0.01 µg per µl RNase A (QIAGEN) for 0, 5, 10, 20, 30, or 45 min at 37°C. Five µl of siRNA-dendrimer complexes at each condition were added to 1.875 µl 1% SDS solution on ice and then subjected to electrophoresis in 1.2% agarose gel and standard TAE buffer. The siRNA bands were stained by ethidium bromide and detected by a Kodak EDAS 290 camera.

Size measurement of G5-siRNA complexes

The TLX siRNA aqueous solution was mixed with indicated amount of the dendrimer G_5 aqueous solution at N/P ratio of 5 in the absence or presence of the RGDK peptide. The final concentration of the siRNA was 1 µM. After incubation at 37 °C for 30 min, size measurement was performed using Zetasizer Nano-ZS (Malvern, Ltd. Malvern, U. K.) with a He-Ne ion laser of 633 nm.

Western blot for neuropilin 1

Twenty µg proteins from the whole cell lysates of HEK293T cells and PBT003 cells were used for western blot analysis. Rabbit anti-Neuropilin 1 antibody (1:500; Millipore; Catalog # AB9600), and mouse anti-GAPDH antibody (1:1000; Gene Tex; Catalog # GTX627408) were used.
